# There is no easy fix to peer review but paying referees and regulating the number of submissions might help

**DOI:** 10.12688/f1000research.148985.1

**Published:** 2024-05-02

**Authors:** Mohamed L. Seghier

**Affiliations:** 1 Department of Biomedical Engineering and Biotechnology, Khalifa University of Science and Technology, Abu Dhabi, Abu Dhabi, United Arab Emirates; 2 Healthcare Engineering Innovation Center (HEIC), Khalifa University of Science and Technology, Abu Dhabi, United Arab Emirates

**Keywords:** peer review, research disseminations, referees, publishers, scholarly communication, awards and incentives, professional reviewers

## Abstract

The exponential increase in the number of submissions, further accelerated by generative AI, and the decline in the availability of experts are burdening the peer review process. This has led to high unethical desk rejection rates, a growing appeal for the publication of unreviewed preprints, and a worrying proliferation of predatory journals. The idea of monetarily compensating peer reviewers has been around for many years; maybe, it is time to take it seriously as one way to save the peer review process. Here, I argue that paying reviewers, when done in a fair and transparent way, is a viable solution. Like the case of professional language editors, part-time or full-time professional reviewers, managed by universities or for-profit companies, can be an integral part of modern peer review. Being a professional reviewer could be financially attractive to retired senior researchers and to researchers who enjoy evaluating papers but are not motivated to do so for free. Moreover, not all produced research needs to go through peer review, and thus persuading researchers to limit submissions to their most novel and useful research could also help bring submission volumes to manageable levels. Overall, this paper reckons that the problem is not the peer review process per se but rather its function within an academic ecosystem dominated by an unhealthy culture of ‘publish or perish’. Instead of reforming the peer review process, academia has to look for better science dissemination schemes that promote collaboration over competition, engagement over judgement, and research quality and sustainability over quantity.

## Introduction

The peer review process has been the cornerstone of science dissemination for centuries. ^1^^,^^2^ It can exist in different forms, ^3^^,^^4^ all based on the elusive concepts of referees’ impartiality ^5^ and competency. ^6^ The peer review process is known to be slow, expensive, inconsistent, biased, ^3^^,^^7^^–^^11^ and with limited impact on the quality of research publications. ^12^^,^^13^ These limitations have led to some calls to completely abandon the whole process because “ *prepublication peer review is an enormous sink of scientists’ time, effort, and resources*”. ^14^ Despite the limitations, one can note that the research community has still strong faith in it ^7^^,^^15^; i.e. “ *despite the limitations of peer review process, we need it. It is all we have, and it is hard to imagine how we could get along without it*”. ^16^ How to reform the peer review process has always been an extremely complex endeavor. ^17^

Many calls have been made to reform the peer review process, typically along four schools of thought according to Waltman (2023). Here, this review subscribes to the Efficiency & Incentives school that focuses on streamlining the peer review process and incentivizing participation in it. ^18^ This is a completely pragmatic choice because without an efficient peer review system there is no point in my opinion of discussing its quality, reproducibility, transparency or inclusion. Those qualities, promoted by other schools of thoughts, are extremely important but they are contingent to the suitability of the existing system to cope with current research productivity levels of millions of scientific articles. ^19^ Specifically, over 2.5 million papers are published each year in English alone, ^20^ creating an incredible burden on editors and reviewers. The problem is obvious to all stakeholders: the exponential increase in the number of manuscripts largely surpasses the current availability of qualified referees. Put another way, the current peer review process is not well equipped to serve modern publication dynamics that are expected to accelerate even more in this era of generative AI.

It might be useful to contemplate what could be the alternative if no adequate and sustainable solutions are found. As nature abhors a vacuum, if the current peer review model is not reformed, frustrated authors will have to rely on other means to get their studies out, in particular when desk rejection rates in reputable journals are reaching high rates. This might for instance lead to a surge in alternative models based on the publication of unreviewed preprints, or the creation of local journals or repositories owned and managed by the authors’ universities and research institutions. ^21^ However, the biggest risk is the undesirable proliferation of predatory journals offering a “pay-to-publish” model sometimes without peer review. ^22^^–^^24^ I believe that models based on pre-publication peer-review are still useful as they do serve the interest of science relatively better than other models. In that context, I discuss here the idea of promoting the role of professional reviewers, like professional editors, who will be monetarily compensated for their role in the peer-review process.

## No more motivation to review for free

Below, I describe a concrete situation based on my own experience as editor-in-chief of a scientific journal. The journal I’m editing, as it might be the case for other journals, is experiencing a (welcome) growth in number of submissions that is putting too much pressure on an already overstretched peer review process. ^25^ Consequently, this situation has resulted in (1) an unethical increase in desk rejection rates, (2) a low percentage of invited researchers willing to review papers, (3) an increase in the number of papers rejected after being sent for peer-review due to the difficulty to secure two or more referee reports, (4) referees not submitting their reports after initially agreeing to do so, (5) referees submitting scant reports, listing superficial, unhelpful, or even irrelevant comments and suggestions, and (6) an increase in instances of fraud where for example false contact information of potential referees with non-institutional emails are suggested or when the peer review process has been manipulated. All these issues are directly or indirectly related to the pressure to ensure fair and rigorous peer review process when the main players (reviewers) are no longer motivated to give away their time and expertise for free.

In this context, a wide range of solutions and innovations have been proposed in the past (see analytical review in ^26^) to address the non-availability of volunteer peer reviewers. ^26^^–^^30^ Table 1 provides a succinct summary of some proposed innovations in current literature. This includes for instance a more efficient involvement of editors in the selection of prospective reviewers, ^31^ improving diversity, ^32^ training reviewers and opening up the peer review process. ^7^ Others have argued that peer reviewers are rejecting invitations not necessarily because of lack of time but presumably because of lack of motivation. ^33^ Therefore, rewarding reviewers can help increase thier (extrinsic) motivation, like offering certificates, discounts on publisher’s products, and publicly acknowledging the best and the most productive reviewers. ^33^^,^^34^

**Table 1.** Previously suggested solutions for improving the peer-review process. A list of suggested solutions and innovations in previous studies. Paying professional reviewers can be implemented in conjunction with any of the solutions below.

Proposed solution	Rationale and description
Implement peer review as an open, continuous, interactive forum involving all stakeholders	This solution is based on two assumptions: (1) there is no evidence that blinding reviewers to the identity of authors would improve the quality process, ^65^ and (2) peer review should promote engagement, not judgement. ^66^ Practically, the idea is to transform the peer review to an open and interactive scientific discussion ^3^^,^^7^ that operates in real time where concerned parties (authors, reviewers, editors) can interact via an open platform. This could involve diverse stakeholders ^8^ through multiple platforms such as blogs and social media. ^1^ This could be seen as one model of the open peer review framework ^67^). Ultimately, this model would promote constructive discussion to enable transparent editorial decisions that aggregate diverse opinions. ^68^
Put in place a hybrid two-tier system where only impactful research is sent for peer review	This solution posits that not all submissions are ‘worth’ going through the prepublication peer review process. Instead, a hybrid model with a two-tier system is applied ^64^: all submissions can be published and subjected to a post-publication peer review, but the prepublication peer review process needs only to evaluate manuscripts that are expected to have a significant impact on the field. ^64^ Basically, this is similar to some extent to desk-rejection practices where only submissions with high novelty and significance are sent for review. The main concern is of course how to make the initial evaluation about impact as objective as possible. This model, without safeguards, can end up biased toward top labs and renowned researchers. Moreover, it is not clear how citations from the unreviewed papers should be considered in the different research metrics.
Offer a non-selective review model to speed up the peer review process	Reviewers are often asked to make judgement about other aspects of the paper beyond its scientific merit, including for instance rating its novelty, significance, impact, originality, readability, and even whether or not the paper will be highly cited if accepted. The non-selective review model mainly focuses on papers’ scientific quality rather than their perceived importance and novelty. ^8^ Therefore, peer reviewers only have to evaluate the methodological soundness of papers. It is also possible to go further along this model by asking reviewers only to review the methods and results section. The other sections (including the authors’ own interpretations of the results in the Discussion section) can be open for discussion after publication.
Involve independent peer review platforms	An initial evaluation of papers quality is performed by other players. It is not unusual to hear peer reviewers complaining about the fact that they are often asked to review papers of poor quality. They believe it is not their job to improve papers as this should be the task of the original authors. In this era of publish as many and as fast as possible, some submissions poorly report the undertaken work, which might make the work of reviewers even harder. Here the idea is to involve other parties to help the authors improve their submissions before submitting to a journal. This has been tested with the creation of independent peer review platforms, ^3^ helping researchers getting their papers in good shape (for example, see platforms Rubriq and Editage). The authors pay the independent reviewers for their contribution in improving their papers. This model might add extra costs to the preparation process of papers.
Adopt a portable peer review across journals and publishers	This idea has been implemented by different initiatives (e.g. the Neuroscience Peer Review Consortium, the Review Commons). It is basically based on having a ‘portable’ peer review across publishers. ^69^ In this model, a paper goes through a journal-independent peer review, then the authors revise their paper accordingly and submit both their revised paper and the review reports to any journal that is taking part in the portable peer-review initiative. The revised submission can be moved between journals until a final editorial decision is made. ^30^ This model might help editors use reviewers time in a more efficient way but might add some delays to the publication of papers (e.g. a paper might remain stuck in the process until it is selected by a suitable journal).
Merge journals into mega-journals with large editorial boards	A mega-journal (like PLOS One and Nature’s Scientific Reports) has a very broad scope, accepting articles in almost all domains. It typically includes a large editorial board and a very large pool of reviewers. Having relatively low rejection rates, mega-journals usually publish thousands of articles per year. They tend to have an efficient (fast turnaround time) peer review process that can handle large submission volumes. ^3^ They sometimes play a similar role as a cascade journal, publishing papers rejected by more selective top journals. ^70^ Because they rely on a large number of academic editors across multiple domains, they are able to involve peer reviewers in an efficient way. However, this mega-journal model might not serve the interests of all domains as specialized journals are needed.
Build an online reviewer registry accessible by all journals	The idea here is to build a large online registry of volunteer reviewers ^71^ who can be invited by any journal that has access to the registry. This registry can be created in a collaborative way by different journals or publishers, including for instance consortia of journals with overlapping scope or domains. ^71^ Such registries can complement the reviewers databases that journals hold. The registry can include reviewers from diverse geographical locations, demographics and expertise. To sustain such initiatives, a cross-publisher partnership has to oversight the creation and the maintenance of the registry, with the mission to support it with the right resources.
Augment the peer process with technology	Technology has already modernised the peer review process ^72^; e.g. automated detection of flawed methods or unethical practices like inappropriate statistical analysis, figure manipulation or data fabrication. There is also a new trend in the development of powerful and versatile software and platforms to streamline the peer-review process (e.g. Scholastica). It is expected that the adoption of generative AI will have many ramifications on the peer review process in the near future. ^73^^,^^74^
You review as much as you publish	As the number of prolific (very productive) researchers is growing very fast, ^63^ this model requires an active involvement from such frequent users of the system. Therefore, researchers who publish the more should contribute the more to the peer review process. ^75^ In fact, it is not unusual to see many senior authors who publish a lot (and hence who are putting too much pressure on the system) are not Involved at all in the per review process. Some have even suggested that senior authors must provide evidence of their contribution to the peer review process as a requirement for considering their manuscripts for review. ^75^ This idea, albeit attractive, might not be feasible as it ignores the fact that senior authors are also very often involved in other academic review duties (doctoral theses, grants applications).
Offer incentives and rewards to reviewers	There is already a variety of rewards offered to reviewers as a recognition of their contribution to the peer review process. For instance, this includes prizes, discounts on publication fees, free access to the publisher’s products (e.g. databases), vouchers for books purchase, reduced registration fees for conferences, recognition in databases (like Publons), and many others. Moreover, active reviewers who are submitting quality reports can be added to the editorial board of the concerned journals. Despite these many incentives, one cannot deny that the number of available reviewers is still declining.
Link peer review involvement to the researcher’s own academic benefits	Other incentives argue for linking promotion applications (e.g. for tenured positions) to how active the researcher is in the peer review process. ^76^ Other suggestions include the possibility to earn continuing education credits upon completion of a review or waived publication fees in the journal. ^77^ Others have even suggested the idea of offering co-authorship to reviewers, ^78^ in particular when the reviewers’ suggestions had significantly improved the original submission.
Abandon the prepublication peer review model and adopt the post-publication peer-review (PPPR) model	The post-publication peer-review (PPPR) model ^79^^,^^80^ can ease the burden on journals with large submission volumes. It does not consider rejection after peer-review. There is already a rich literature about this model, ^67^ and it has been tested experimentally by different initiatives (e.g. F1000Research, ScienceOpen, The Winnower). Despite its merits, this model has some limitations as it might let all papers to be available in the public domain, regardless of their original quality and impact. Without safeguards and an initial thorough check of paper quality, this model might end up mimicking the work of predatory journals that publish all submissions without (prepublication) peer review. Hence, it important that PPPR allows for the continuous discussion and improvement of the quality of science. ^79^ However, there has to be a limit on when a paper can still remain open for discussion as progress in science will lead to new experimental protocols and novel conceptual frameworks that will make ‘old’ publications look erroneous or obsolete.
Abandon the peer review process altogether	The (prepublication) peer review process should be abandoned as there is no strong evidence about its usefulness (e.g. see discussion in Ref. 14). The alternative is to gradually move toward expanding the role of preprint servers ^8^^,^^21^ or by allowing universities and research institutions to manage the publications made by their researchers. It is the researchers who create papers and it is thus the responsibility of their institutions to disseminate their work in the format they deem fit.

However, these suggestions might not solve existing problems for four main reasons: (i) the number of submissions is projected to increase at much faster rates in this era of generative AI, (ii) the traditional job of academia is being transformed with extra roles that researchers are expected to assume, leaving no time for reviewing papers, including admin duties, seeking funds, leadership roles, academic advising, organizing outreach activities in the community, and working on translating their research into marketable solutions, (iii) the number of journals and publications in other languages than English is growing fast, promoted by current initiatives to improve inclusion in science dissemination, which makes it challenging to ensure a rigorous multilingual peer review process, (iv) researchers are increasingly unsatisfied with for-profit publishers that have made exorbitant profits with negligible contribution to research in general, resulting in many researchers not keen to review for for-profit journals, and (v) perhaps most importantly, researchers are likely to become less willing to spend time on reviewing AI-enhanced quick-to-write papers (e.g. when the time taken to review a paper becomes longer than the time taken to draft that paper with AI). I believe the latter is likely to be increasingly present in the debate about reforming the peer review process, which is why monetary incentives might be one potential means to alleviate the burden on the peer review process to some extent.

## Toward the promotion of Professional Reviewer as a career

Publishing research articles is still an expensive business even in this digital era. ^35^^–^^37^ The global annual cost of peer-review is estimated at around $1.5 billion, ^38^ corresponding to a net annual contribution of over 100 million hours by reviewers. ^39^ In the current science dissemination business evaluated at billions of dollars, peer reviewers are still providing volunteer labour. Journals should thus develop methods to compensate reviewers for their time while maintaining the integrity of the peer-review process. ^38^^,^^40^ The system needs fair and sustainable mechanisms to pay referees. This would allow researchers to earn money by becoming full-time or part-time professional reviewers. Like the remunerated role played by professional language editors, researchers who enjoy evaluating research papers can be invited to dedicate more time to the peer review process for a fee. ^41^ For instance, a professional reviewer can be tasked with reviewing a given number of papers per week (e.g. 2 or 3 papers/week), which would help speed up the peer review process in particular for journals with large submission volumes.

**Figure 1.**
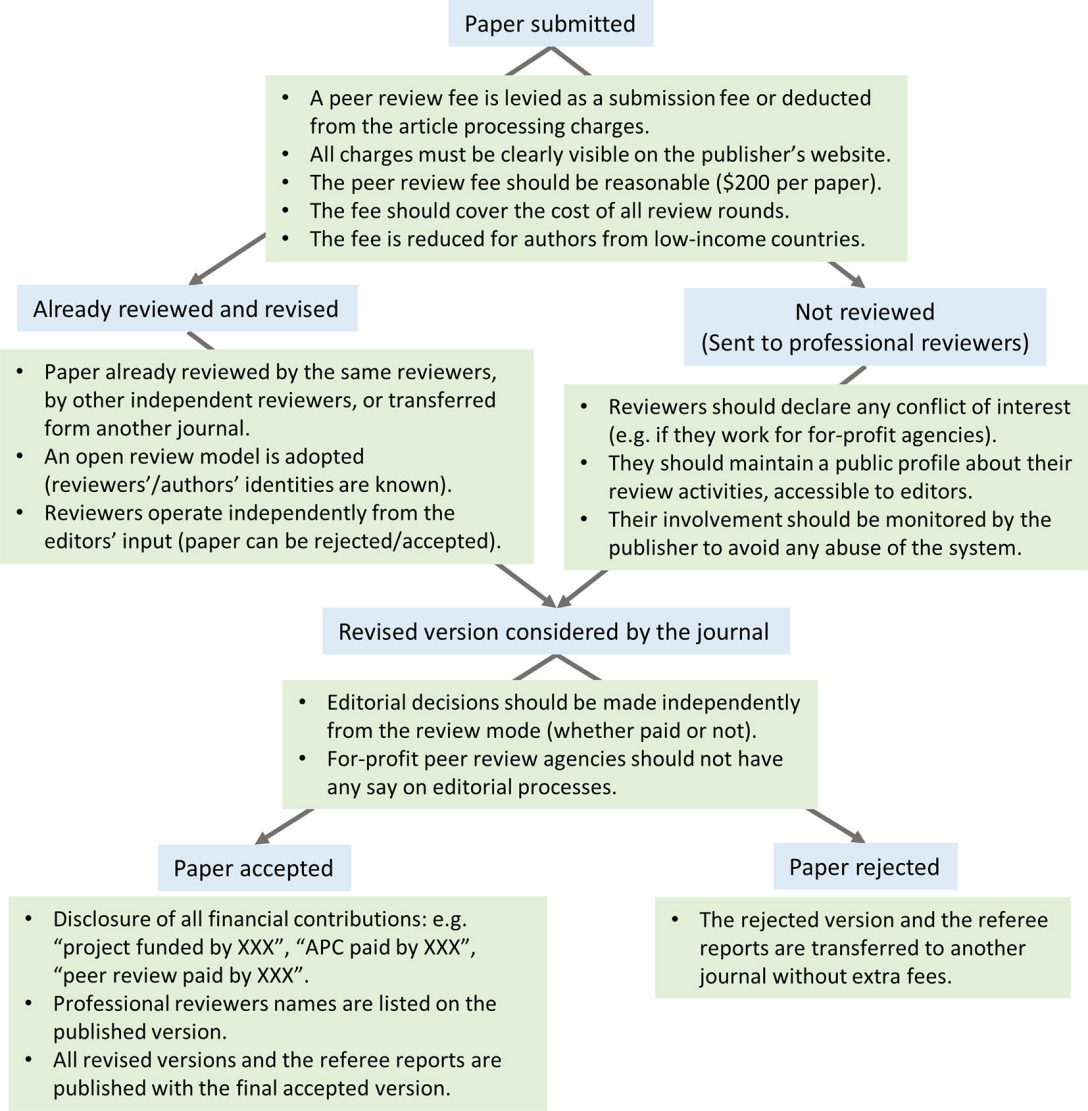
Professional reviewers can be an integral part of the peer review process. A typical publication process that involves professional reviewers. It shows the different steps (blue boxes) and their implementation in a fair and transparent way (green boxes).

We already have models where referees are paid for reviewing academic work, as grant reviewers, PhD examiners, or academic promotion examiners. Reviewing articles is no exception and could thus be considered as a remunerated task in academia. There is room for the emergence of two different but complementary professional reviewers. Reviewers can still be academicians, working in universities and research institutions, where they can be offered the opportunity to supplement their salaries with additional honorariums for their active involvement in the peer review process. Another alternative concerns the possibility to outsource the peer review process by allowing the creation of independent providers. These providers, managed by for-profit companies, ^42^ can follow the same work model as companies that employ professional language editors in research dissemination. The job of a professional reviewer can be regulated by adopting new ethical and professional standards to safeguard the peer review process from any possible unethical practices that might occur when money is involved in the process ( Figure 1).

The proponent of this idea argues that per review should be considered as a business transaction, ^43^ where a modest remuneration per paper of around 200$ ^43^, or $450 for for-profit publishers, ^44^ can be offered to reviewers. This can motivate reviewers to be more actively involved in the peer review process and to offer fast and thorough review reports. ^45^^,^^46^ This can also be an incentive for retired scientists ^43^^,^^47^ to participate in order to gain an extra income that expands their retirement plans. Paying referees can increase the pool of available reviewers, including for instance researchers who cannot afford to work for free. ^47^ Below, I discuss some options to sustain the payment of professional referees.

***Reviewers as consultants:*** Researchers are usually invited to budget for the support of a consultant or a technical expert when applying for grants. For instance, the consultant can provide expert opinion about an aspect of the research project that might be beyond the expertise of the researchers (e.g. a legal advisor, an industrial partner, a clinical professional, a computer system expert, a programmer, … etc). In the same way, a peer reviewer can be seen as a consultant whose role is to support the researchers in improving their papers in terms of readability, quality, and methodological soundness. Publications are typically included as deliverables in research grants, and therefore the contribution of peer reviewers to improve these deliverables can be budgeted as consultancy fees. However, this option might put too much financial burden on funding bodies as they are already covering the excessive article-processing charges of publications. Similar to some alternatives being sought for article-processing charges payments, ^48^ funding bodies should also work closely with publishers to make the payment process of professional reviewers as fair and transparent as possible, in particular when for-profit agencies are managing the peer review process.

***Levying a submission fee:*** As it is practice in some journals, a submission fee can be collected for each submitted article, which can then be used to pay professional reviewers. For example, around 45 of the economics, finance and accounting journals published by Elsevier charge a submission fee of around $50-$100. Likewise, some biology journals also levy submission fees. Their rationale is to ensure that only within-scope and high-quality papers are received. Submission fees also serve other purposes like sponsoring conferences and workshops, the payment of language editing services, prizes/awards such as the annual best paper award, and even payments to top reviewers. To make payment of professional reviewers sustainable, it might be suggested to levy a submission fee of $200 per submitted paper. This fee should only be charged for submissions sent for review. I believe a $200 would be a fair submission fee for the service of getting constructive comments about how to improve a paper. For example, a journal with a submission volume of 1,000 papers per year, and a desk-rejection rate of 50%, the collected submission fees (around $100,000) can help secure the services of professional reviewers. To make this option attractive for authors, the submission fee should be deduced from the article-processing charges or the open access fee once the peer-reviewed paper is accepted. Consequently, this might mean that publishers should consider reducing their article processing charges when levying a submission fee, as discussed below.

***Paying referees in the context of economic disparity:*** One of the consequences of paying reviewers might be a better diversity in peer review. This is because paying referees might be very appealing for researchers from low- or middle-income countries. However, this monetary incentive can inadvertently yield over-involvement in the peer review process because a fee of 200$ or more is sometimes comparable to a full salary in some low-income countries. Put another way, researchers from low-income countries can favour paid peer review over other academic duties, which might not be a tenable situation for their institutions. One way to avoid this race for more reviews, and hence more money, is to link the number of invitations for referees from low-income countries to the size of authors or readers from those low-income countries. For instance, if a journal has a readership of 20% from low-income countries, the number of invited referees from low-income countries can be capped at twice this percentage. Furthermore, all paid review tasks should be declared on the publisher’s website so that editors are well informed about the level of involvement of different referees. This would help identify any unusual review dynamics that need adjustments to ensure a fair and robust peer review.

***How about the publishers?*** There is a clear dissatisfaction from academia about the ‘parasitic’ behaviour of some for-profit publishers in research dissemination. They are seen as the biggest winners of the current system despite not funding research and not paying the original content creators (the authors) and evaluators (the reviewers). ^49^ Recent examples of editors quitting for-profit journals provide an interesting example to grasp with the depth of that dissatisfaction (e.g. Refs. 50, 51). My own reading of the publication landscape makes me believe that for-profit publishers, if they do not engage with the research community in a more honest and constructive way, will soon be made irrelevant to the whole process. It is important that publishers realize that they cannot continue operating along the same model. The least one can expect from them is to inject some of their profits back to the peer review process. However, it is unlikely that publishers would be willing to financially contribute to the peer review process unless they have control on the process through the creation of their own professional review agencies. However, I don’t think the research community can again trust for-profit publishers with the peer review process, which is understandable given the previous experience of the community with the open access model that has unfortunately been made so expensive and unaffordable by some for-profit publishers.

In this context, I feel that research dissemination might foreseeably follow a radical model by completely bypassing for-profit publishers, i.e. a model that does not need the ‘middleman’. This implies that researchers and their universities will soon own the whole process of research dissemination. ^52^ In this model, universities and research institutions might chose to manage the whole publication process, for instance in the form of posting papers on open repositories, ^53^ while adopting an open post-publication peer review model. Universities can work as consortia to share the cost of publication through common or shared repositories. The other implication here is to allow researchers (or their institutions) keep intellectual property of their work instead of conceding it to publishers as in the current model.

***Arguments against paying peer reviewers:*** The opponents to paying reviewers mention the risks of proliferation of unethical practices (i.e. the system to be corrupted by money) and unsustainable costs ^44^^,^^47^^,^^54^ as it can lead to increases in subscription fees and article processing charges. ^55^ Moreover, adding a monetary component to the peer review might lead to unethical practices to maximize the number of completed reviews such as non-disclosure of conflict of interest, the tendency to review papers even outside one’s domain of expertise, and a fast turnaround time with no through evaluation of the reviewed papers. Therefore, it is important to put some safeguards to ensure high quality and rigorous peer review process, for instance, through the evaluation/rating by the editors of completed reviews before approving payment to the referees.

Likewise, paying reviewers might yield a proliferation of for-profit peer review agencies with dubious ethical practices, like paper mills, that can generate fabricated review reports. ^55^ Some studies have argued that monetary rewards tend to decrease the quality of the peer review process ^56^ as well as the intrinsic motivation of researchers to contribute to the peer review process. ^57^ Paying reviewers might be only sustainable for journals with a reasonable submission volume. ^58^ For multidisciplinary journals with broad topics and large submission volumes, the peer review process can be enormously costly and thus diverse funding sources have to be sought. There is also the risk that paying referees will soon become the norm, making researchers no longer interested to review for free. It could also lead to a multi-tier peer review system depending on how much a journal chose to pay its reviewers, hence engendering an unequal competition between journals for the service of reviewers. Overall, although one cannot rule out the risk of corruption in peer review by money, I trust that academia has in-built mechanisms to handle financial transactions in a fair and objective way as it is the case when researchers are paid for evaluating grant proposals and academic theses. ^47^

***Other schemes of financial compensations:*** It might be worth mentioning other alternative models to professional reviewers. For example, one proposal argues for offering monetary compensations not for the reviewers themselves but their institutions ^59^ as they usually deal with the same publishers for purchasing subscriptions and other products. The rationale is that reviewers are already paid by their institutions and hence their involvement in the peer review process should benefit their institutions. However, this sometime ignores the fact that researchers sometimes (often?) conduct their reviews outside work hours (e.g. evenings or weekends), and hence their contributions to peer review should not be assumed already covered by their salaries. Likewise, another similar recent proposal argues for monetary prizes not to individual referees but to their groups (departments, research units or labs). ^57^ That collected money for instance can support ongoing research projects, support early career members or the work of members from underrepresented minorities. ^57^

## Peer review cannot thrive in the current academic ecosystem

The above-mentioned solutions are assumed remedies to a malfunctional peer review process. But maybe the whole peer review process is fine, and all encountered problems are due to the fact that peer review is operating within the wrong environment. Indeed, the culprit here is the current academic environment that promotes an unhealthy culture of “publish or perish”. To survive in this culture, it is not unusual that researchers do fragment their results into multiple publications or even publish redundant papers based on similar data. ^30^^,^^60^ This culture of “publish or perish” is hurting academic research. ^61^ Therefore, instead of instigating calls to abandon or reform the peer review process, academia should invent and campaign for new models that departs from the “publish or perish” model. ^62^ As long as research quality is measured as a function of research productivity, there will be no form of peer review that can guarantee quality research while dealing with a deluge of papers that is now being inflated even more by the adoption of generative AI.

Is the ultimate goal of academia to produce prolific researchers who can publish two or more papers a week, sometimes with questionable practices. ^63^ If, hypothetically, this goal is achieved by every researcher, who would review (and read) the published work? In fact, evidence shows that many published papers remain uncited or end up having little impact on their respective fields, but one cannot ignore the resources those papers took (time and effort) from reviewers and editors. It is as if the whole purpose of many substandard papers is to inflate some research metrics. Can academia promote a new model that puts a cap on how many papers an author can submit per year? This is essentially to invite researchers to think sensibly about the quality of their research and maybe only publish the best of their research that is likely to have an impact on the knowledge creation enterprise. Other work can still be shared as unreviewed preprints. ^64^ This framework ensures a manageable number of submissions, though it might entail a rethinking of current citation-based metrics of research impact and productivity.

## Conclusion

We can’t afford to ignore existing flaws in the current peer review system because the *status quo* will only favour the emergence of unethical practices. Here, I support the idea, albeit not new, of the creation of professional peer reviewers. Paying reviewers must be made in a fair and transparent way. Future work needs to investigate alternative ways to ensure a sustainable and affordable remuneration of professional reviewers. The discussion should continue among all stakeholders to identify new solutions and innovations for an efficient and affordable research dissemination in the modern era of generative AI. Empowering universities and research institutions to own the whole research dissemination system might soon be an inevitable scheme to consider. Finally, without reforming academia when it comes to disseminating the work of its members, research dissemination will continue relying on a costly and ineffective peer review system that will soon or later be completely abandoned or replaced by something else.

## Contributions

This opinion article was conceived and written by MLS.

## Data Availability

No data are associated with this article.

## References

[1] Castillo M : Peer review: past, present, and future. *AJNR Am. J. Neuroradiol.* 2012;33(10):1833–1835. 10.3174/ajnr.A3025 22403775 PMC7964625

[2] Spier R : The history of the peer-review process. *Trends Biotechnol.* 2002;20(8):357–358. 10.1016/S0167-7799(02)01985-6 12127284

[3] Jubb M : Peer review: The current landscape and future trends. *Learn. Publ.* 2016;29(1):13–21. 10.1002/leap.1008

[4] Koshy K Fowler AJ Gundogan B : Peer review in scholarly publishing part A: why do it? *Int. J. Surg. Oncol.* 2018;3(2):e56. 10.1097/IJ9.0000000000000056

[5] Atkinson M : Regulation of Science by “Peer Review”. *Stud. Hist. Phil. Sci.* 1994;25:147–158. 10.1016/0039-3681(94)90025-6

[6] Baxt WG Waeckerle JF Berlin JA : Who reviews the reviewers? Feasibility of using a fictitious manuscript to evaluate peer reviewer performance. *Ann. Emerg. Med.* 1998;32(3 Pt 1):310–317. 10.1016/S0196-0644(98)70006-X 9737492

[7] Smith R : Peer review: a flawed process at the heart of science and journals. *J. R. Soc. Med.* 2006;99(4):178–182. 10.1177/014107680609900414 16574968 PMC1420798

[8] Walker R Rocha da Silva P : Emerging trends in peer review-a survey. *Front. Neurosci.* 2015;9:169. 26074753 10.3389/fnins.2015.00169 PMC4444765

[9] Haffar S Bazerbachi F Murad MH : Peer Review Bias: A Critical Review. *Mayo Clin. Proc.* 2019;94(4):670–676. 10.1016/j.mayocp.2018.09.004 30797567

[10] Rooyen S van : A critical examination of the peer review process. *Learn. Publ.* 1998;11(3):185–191. 10.1087/09531519850146355

[11] Williamson A : What will happen to peer review? *Learn. Publ.* 2003;16(1):15–20. 10.1087/095315103320995041

[12] Jefferson T Wager E Davidoff F : Measuring the quality of editorial peer review. *JAMA.* 2002;287(21):2786–2790. 10.1001/jama.287.21.2786 12038912

[13] Wager E Jefferson T : Shortcomings of peer review in biomedical journals. *Learn. Publ.* 2001;14(4):257–263. 10.1087/095315101753141356

[14] Heesen R Bright LK : Is Peer Review a Good Idea? *Br. J. Philos. Sci.* 2021;72(3):635–663. 10.1093/bjps/axz029

[15] Jefferson T Alderson P Wager E : Effects of editorial peer review: a systematic review. *JAMA.* 2002;287(21):2784–2786. 10.1001/jama.287.21.2784 12038911

[16] Relman AS : Peer review in scientific journals--what good is it? *West. J. Med.* 1990;153(5):520–522. 2260288 PMC1002603

[17] Chloros GD Giannoudis VP Giannoudis PV : Peer-reviewing in Surgical Journals: Revolutionize or Perish? *Ann. Surg.* 2022;275(1):e82–e90. 10.1097/SLA.0000000000004756 33630457

[18] Waltman L Kaltenbrunner W Pinfield S : How to improve scientific peer review: Four schools of thought. *Learn. Publ.* 2023;36(3):334–347. 10.1002/leap.1544 38504796 PMC10946616

[19] Jinha AE : Article 50 million: an estimate of the number of scholarly articles in existence. *Learn. Publ.* 2010;23(3):258–263. 10.1087/20100308

[20] Tennant JP : The state of the art in peer review. *FEMS Microbiol. Lett.* 2018;365(19). 10.1093/femsle/fny204 30137294 PMC6140953

[21] Gogotsi Y : Pay to publish? Open access publishing from the viewpoint of a scientist and editor. *Graphene 2D Mater.* 2023;8:1–3. 10.1007/s41127-023-00057-3

[22] Camargo LM Smirnov M Maldonado IL : The prey’s perspective on the rise of predatory publishing. *EXCLI J.* 2023;22:904–906. 10.17179/excli2023-6392 37780943 PMC10539542

[23] Grudniewicz A Moher D Cobey KD : Predatory journals: no definition, no defence. *Nature.* 2019;576(7786):210–212. 10.1038/d41586-019-03759-y 31827288

[24] Mertkan S Onurkan G Nilgun Suphi G : Profile of authors publishing in ‘predatory’ journals and causal factors behind their decision: A systematic review. *Res. Eval.* 2021;30(4):470–483. 10.1093/reseval/rvab032

[25] Seghier ML : Demystifying desk rejection: A call to action for our authors. *Int. J. Imaging Syst. Technol.* 2022;32(3):701–703. 10.1002/ima.22733

[26] Kaltenbrunner W Pinfield S Waltman L : Innovating peer review, reconfiguring scholarly communication: an analytical overview of ongoing peer review innovation activities. *J. Doc.* 2022;78(7):429–449. 10.1108/JD-01-2022-0022

[27] Woods HB Brumberg J Kaltenbrunner W : An overview of innovations in the external peer review of journal manuscripts. *Wellcome Open Res.* 2022;7:82. 10.12688/wellcomeopenres.17715.1 36879926 PMC9984734

[28] Spier RE : Peer review and innovation. *Sci. Eng. Ethics.* 2002;8(1): p.99–108. discussion 109-12. 10.1007/s11948-002-0035-0 11840960

[29] Barroga E : Innovative Strategies for Peer Review. *J. Korean Med. Sci.* 2020;35(20):e138. 10.3346/jkms.2020.35.e138 32449322 PMC7246191

[30] Stahel PF Moore EE : Peer review for biomedical publications: we can improve the system. *BMC Med.* 2014;12:179. 10.1186/s12916-014-0179-1 25270270 PMC4177268

[31] Zupanc GKH : “It is becoming increasingly difficult to find reviewers”—myths and facts about peer review. *J. Comp. Physiol. A.* 2023;210:1–5. in press. 10.1007/s00359-023-01642-w PMC10266957 37318565

[32] Ben Messaoud K Schroter S Richards M : Analysis of peer reviewers’ response to invitations by gender and geographical region: cohort study of manuscripts reviewed at 21 biomedical journals before and during covid-19 pandemic. *BMJ.* 2023;381:e075719. 10.1136/bmj-2023-075719 37311585 PMC10471900

[33] Ellwanger JH Chies JAB : We need to talk about peer-review-Experienced reviewers are not endangered species, but they need motivation. *J. Clin. Epidemiol.* 2020;125:201–205. 10.1016/j.jclinepi.2020.02.001 32061827

[34] Künzli N Berger A Czabanowska K : «I Do Not Have Time»—Is This the End of Peer Review in Public Health Sciences? *Public Health Rev.* 2022;43:1605407. 10.3389/phrs.2022.1605407 36467128 PMC9716458

[35] Grossmann A Brembs B : Current market rates for scholarly publishing services [version 2; peer review: 2 approved]. *F1000Res.* 2021;10:20. 10.12688/f1000research.27468.1 34316354 PMC8276192

[36] Smart P : Peer review: An expensive business. *Learn. Publ.* 2016;29:3–4. 10.1002/leap.1012

[37] Donovan B : The truth about peer review. *Learn. Publ.* 1998;11(3):179–184. 10.1087/09531519850146346

[38] LeBlanc AG Barnes JD Saunders TJ : Scientific sinkhole: estimating the cost of peer review based on survey data with snowball sampling. *Res. Integr. Peer Rev.* 2023;8(1):3. 10.1186/s41073-023-00128-2 37088838 PMC10122980

[39] Aczel B Szaszi B Holcombe AO : A billion-dollar donation: estimating the cost of researchers’ time spent on peer review. *Res. Integr. Peer Rev.* 2021;6(1):14. 10.1186/s41073-021-00118-2 34776003 PMC8591820

[40] Gasparyan AY Gerasimov AN Voronov AA : Rewarding peer reviewers: maintaining the integrity of science communication. *J. Korean Med. Sci.* 2015;30(4):360–364. 10.3346/jkms.2015.30.4.360 25829801 PMC4366954

[41] Ott S Hebenstreit D : Supply and demand: Apply market forces to peer review. *Nature.* 2014;506(7488):295. 10.1038/506295b 24553233

[42] Van Noorden R : Company offers portable peer review. *Nature.* 2013;494(7436):161. 10.1038/494161a 23407520

[43] Diamandis EP : Publishing costs: Peer review as a business transaction. *Nature.* 2015;517(7533):145. 10.1038/517145a 25567274

[44] Brainard J : The $450 question: Should journals pay peer reviewers? *Science.* 2021. 10.1126/science.abh3175

[45] Hamermesh DS : Facts and Myths about Refereeing. *J. Econ. Perspect.* 1994;8(1):153–163. 10.1257/jep.8.1.153

[46] Thompson GD Aradhyula SV Frisvold G : Does Paying Referees Expedite Reviews?: Results of a Natural Experiment. *South. Econ. J.* 2010;76(3):678–692. 10.4284/sej.2010.76.3.678

[47] Cheah PY Piasecki J : Should peer reviewers be paid to review academic papers? *Lancet.* 2022;399(10335):1601. 10.1016/S0140-6736(21)02804-X 35461548

[48] Sanderson K : Who should pay for open-access publishing? APC alternatives emerge. *Nature.* 2023;623(7987):472–473. 10.1038/d41586-023-03506-4 37964063

[49] Walter P Mullins D : From symbiont to parasite: the evolution of for-profit science publishing. *Mol. Biol. Cell.* 2019;30(20):2537–2542. 10.1091/mbc.E19-03-0147 31539315 PMC6740196

[50] Sanderson K : Editors quit top neuroscience journal to protest against open-access charges. *Nature.* 2023;616(7958):641. 10.1038/d41586-023-01391-5 37085706

[51] Singh Chawla D : Open-access row prompts editorial board of Elsevier journal to resign. *Nature.* 2019. 10.1038/d41586-019-00135-8

[52] Roh C : Owning the peer review process. *Coll. Res. Libr. News.* 2022;83(3). 10.5860/crln.83.3.100

[53] Torres L Hartley R : Repositories for academic products/outputs: Latin American and Chilean visions. *F1000Res.* 2019;8:1517. 10.12688/f1000research.19976.1 31700616 PMC6820816

[54] Gropp RE Glisson S Gallo S : Peer Review: A System under Stress. *Bioscience.* 2017;67(5):407–410. 10.1093/biosci/bix034

[55] Moustafa K : No to paid peer review. *Lancet.* 2022;400(10347):160. 10.1016/S0140-6736(22)01057-1 35843244

[56] Squazzoni F Bravo G Takacs K : Does incentive provision increase the quality of peer review? An experimental study. *Res. Policy.* 2013;42(1):287–294. 10.1016/j.respol.2012.04.014

[57] Bonaccorsi A : Towards Peer Review As a Group Engagement. *JLIS. It.* 2022;14(1):46–59. 10.36253/jlis.it-511

[58] Chang J-J Lai C-C : Is It Worthwhile to Pay Referees? *South. Econ. J.* 2001;68(2):457–463.

[59] Copiello S : On the money value of peer review. *Scientometrics.* 2018;115:613–620. 10.1007/s11192-018-2664-3

[60] Beaufils P Karlsson J : Legitimate division of large datasets, salami slicing and dual publication. Where does a fraud begin? *Orthop. Traumatol. Surg. Res.* 2013;99(2):121–122. 10.1016/j.otsr.2013.01.001 23434431

[61] Rawat S Meena S : Publish or perish: Where are we heading? *J. Res. Med. Sci.* 2014;19(2):87–89. 24778659 PMC3999612

[62] Yeo MA Renandya WA Tangkiengsirisin S : Re-envisioning Academic Publication: From “Publish or Perish” to “Publish and Flourish”. *RELC J.* 2022;53(1):266–275. 10.1177/0033688220979092

[63] Ioannidis JPA Collins TA Baas J : Evolving patterns of extremely productive publishing behavior across science. 2023. Preprint. 10.1101/2023.11.23.568476v2

[64] Arns M : Open access is tiring out peer reviewers. *Nature.* 2014;515(7528):467. 10.1038/515467a 25428463

[65] Rooyen S van Godlee F Evans S : Effect of blinding and unmasking on the quality of peer review: a randomized trial. *JAMA.* 1998;280(3):234–237. 10.1001/jama.280.3.234 9676666

[66] Kennison R : Back to the future: (re) turning from peer review to peer engagement. *Learn. Publ.* 2016;29(1):69–71. 10.1002/leap.1001

[67] Ross-Hellauer T : What is open peer review? A systematic review [version 2; peer review: 4 approved]. *F1000Res.* 2017;6:588. 10.12688/f1000research.11369.1 28580134 PMC5437951

[68] Marcoci A Vercammen A Bush M : Reimagining peer review as an expert elicitation process. *BMC. Res. Notes.* 2022;15(1):127. 10.1186/s13104-022-06016-0 35382867 PMC8981826

[69] Bell GP Kvajo M : Tackling waste in publishing through portable peer review. *BMC Biol.* 2018;16(1):146. 10.1186/s12915-018-0619-z 30558673 PMC6297941

[70] Spezi V Wakeling S Pinfield S : Open-Access mega-journals: The future of scholarly communication or academic dumping ground? A review. *J. Doc.* 2017;73:263–283. 10.1108/JD-06-2016-0082

[71] Ling F : Improving peer review: increasing reviewer participation. *Learn. Publ.* 2011;24:231–233. 10.1087/20110311

[72] Papatriantafyllou M : Peer Review - the future is here. *FEBS Lett.* 2017;591(18):2789–2792. 10.1002/1873-3468.12792 28805250

[73] Kousha K Thelwall M : Artificial intelligence to support publishing and peer review: A summary and review. *Learn. Publ.* 2024;37:4–12. (in press). 10.1002/leap.1570

[74] Heaven D : AI peer reviewers unleashed to ease publishing grind. *Nature.* 2018;563(7733):609–610. 10.1038/d41586-018-07245-9 30482927

[75] Graur D : Payback time for referee refusal. *Nature.* 2014;505:483. 10.1038/505483a 24451533

[76] Laxdal A Haugen T : Where are the carrots? A proposal to start crediting peer reviewers for their contribution to science. *Learn. Publ.* 2024;37:154–156. (in press). 10.1002/leap.1589

[77] Gorin MA : Combating reviewer fatigue with carrots. *BJUI Compass.* 2023;4(1):3–4. 10.1002/bco2.207 36569502 PMC9766864

[78] Kumar MN : The ‘peer reviewer as collaborator’ model for publishing. *Learn. Publ.* 2010;23(1):17–22. 10.1087/20100105

[79] Teixeira da Silva JA Dobranszki J : Problems with traditional science publishing and finding a wider niche for post-publication peer review. *Account. Res.* 2015;22(1):22–40. 10.1080/08989621.2014.899909 25275622

[80] Teixeira da Silva JA Al-Khatib A Dobranszki J : Fortifying the Corrective Nature of Post-publication Peer Review: Identifying Weaknesses, Use of Journal Clubs, and Rewarding Conscientious Behavior. *Sci. Eng. Ethics.* 2017;23(4):1213–1226. 10.1007/s11948-016-9854-2 27909954

