# Paying reviewers and regulating the number of papers may help fix the peer-review process

**DOI:** 10.12688/f1000research.148985.4

**Published:** 2025-06-24

**Authors:** Mohamed L. Seghier

**Affiliations:** 1 Healthcare Engineering Innovation Center (HEIC), Khalifa University of Science and Technology, Abu Dhabi, United Arab Emirates; 2 Department of Biomedical Engineering and Biotechnology, Khalifa University of Science and Technology, Abu Dhabi, Abu Dhabi, United Arab Emirates

**Keywords:** peer review, research disseminations, referees, publishers, scholarly communication, awards and incentives, professional reviewers

## Abstract

The exponential increase in the number of submissions, further accelerated by generative AI, and the decline in the availability of experts are burdening the peer review process. This has led to high unethical desk rejection rates, a growing appeal for the publication of unreviewed preprints, and a worrying proliferation of predatory journals. The idea of monetarily compensating scientific peer reviewers has been around for many years; maybe, it is time to take it seriously as one way to save the peer review process. Here, I argue that paying reviewers, when done in a fair and transparent way, is a viable solution. Like the case of professional language editors, part-time or full-time professional reviewers, managed by universities or for-profit companies, can be an integral part of modern peer review. Being a professional reviewer could be financially attractive to retired senior researchers and to researchers who enjoy evaluating papers but are not motivated to do so for free. Moreover, not all produced research needs to go through peer review, and thus persuading researchers to limit submissions to their most novel and useful research could also help bring submission volumes to manageable levels. This paper also reckons that the problem is not the peer review process per se but rather its function within an academic ecosystem dominated by an unhealthy and unsustainable culture of ‘publish or perish’. Instead of reforming the peer review process, academia has to look for better science dissemination schemes that promote collaboration over competition, engagement over judgement, and research quality and sustainability over quantity.

## Introduction

The peer review process has been the cornerstone of science dissemination for centuries. ^1^^,^^2^ It exists in different forms, ^3^^,^^4^ all based on the elusive concepts of referees’ impartiality ^5^ and competency. ^6^ The peer review process is known to be slow, expensive, inconsistent, biased, ^3^^,^^7^^–^^11^ and with presumably limited impact on the quality of research publications. ^12^^,^^13^ These limitations have led to some calls to completely abandon the whole process because “ *prepublication peer review is an enormous sink of scientists’ time, effort, and resources*”. ^14^ Despite the limitations, one can note that the research community has still strong faith in it ^7^^,^^15^; i.e. “ *despite the limitations of peer review process, we need it. It is all we have, and it is hard to imagine how we could get along without it*”. ^16^ How to reform the peer review process has always been an extremely complex endeavor. ^17^^,^^18^

Many calls have been made to reform the peer review process, typically along four schools of thought according to Waltman and colleagues (2023). Here, this paper subscribes to the Efficiency & Incentives school that focuses on streamlining the peer review process and incentivizing participation in it. ^19^ This is a completely pragmatic choice because without an efficient peer review system, there is no point in my opinion in discussing its quality, reproducibility, transparency, or inclusion. Those qualities, promoted by other schools of thought, are extremely important but they are contingent on the suitability of the existing system to cope with the current research productivity levels of millions of scientific articles. ^20^ Specifically, over 2.5 million papers are published each year in English alone, ^21^ creating an incredible burden on editors and reviewers. The problem is obvious to all stakeholders: the exponential increase in the number of manuscripts ^22^^,^^23^ largely surpasses the current availability of qualified referees. Put another way, the current peer review process is not well equipped to serve modern publication dynamics that are expected to accelerate even more in this era of generative AI.

Adequate and sustainable alternative solutions are needed. As nature abhors a vacuum, if the current peer review model is not reformed, frustrated authors will have to rely on other means to get their studies out, in particular when desk rejection is reaching high rates. ^24^^,^^25^ This might for instance lead to a surge in alternative models based on the publication of unreviewed preprints, or the creation of local journals or repositories owned and managed by the authors’ universities and research institutions. ^26^ However, the biggest risk is the undesirable proliferation of predatory journals offering a “pay-to-publish” model sometimes without peer review. ^27^^–^^29^ I believe that models based on pre-publication peer-review are still useful as they do serve the interest of science relatively better than other models. ^18^ In that context, I discuss here the idea of promoting the role of professional reviewers, like professional editors, who will be monetarily compensated for their role in the peer-review process.

## No more motivation to review for free

Below, I describe a concrete situation based on my own experience as editor-in-chief of a scientific journal. The journal I am editing, as might be the case for other journals, is experiencing a (welcome) growth in the number of submissions that are putting too much pressure on an already overstretched peer review process. ^25^ Consequently, this situation has resulted in (i) an unethical increase in desk rejection rates, (ii) a low percentage of invited researchers willing to review papers, (iii) an increase in the number of papers rejected after being sent for peer-review due to the difficulty to secure two or more referee reports, (iv) referees not submitting their reports after initially agreeing to do so, (v) referees submitting scant reports, listing superficial, unhelpful, or even irrelevant comments and suggestions, and (vi) an increase in instances of fraud where for example false contact information of potential referees with non-institutional emails was suggested or when the peer review process was manipulated. ^30^^–^^32^ All these issues are directly or indirectly related to the pressure to ensure a fair and rigorous peer review process when the main players (reviewers) are no longer motivated to give away their time and expertise for free.

In this context, a wide range of solutions and innovations were proposed in the past (see analytical review in ^33^) to address the non-availability of volunteer peer reviewers. ^18^^,^^33^^–^^39^ Table 1 provides a succinct summary of some proposed innovations in the current literature. This includes, for instance, a more efficient involvement of editors in selecting prospective reviewers, ^40^ improving diversity, ^41^ training reviewers, and opening up the peer review process. ^7^ Others have argued that scientific peer reviewers are rejecting invitations not necessarily because of lack of time but presumably because of lack of motivation. ^42^ Therefore, rewarding reviewers can help increase their (extrinsic) motivation, like offering certificates, discounts on publisher’s products, and publicly acknowledging the best and the most productive reviewers. ^42^^,^^43^

**Table 1.** Previously suggested solutions for improving the peer-review process. A list of suggested solutions and innovations in previous studies. Paying professional reviewers can be implemented in conjunction with any of the solutions below.

Proposed solution	Rationale and description
Implement peer review as an open, continuous, interactive forum involving all stakeholders	This solution is based on two assumptions: (1) blinding reviewers to the identity of authors does not improve the quality of peer review, ^87^ and (2) peer review should promote engagement, not judgement. ^88^ Practically, the idea is to transform the peer review into an open and interactive scientific discussion ^3^^,^^7^ that operates in real time where concerned parties (authors, reviewers, editors) can interact via an open platform. This could involve diverse stakeholders ^8^ through multiple platforms such as blogs and social media. ^1^ This could be seen as one model of the open peer review framework ^89^). Ultimately, this model would promote constructive discussion to enable transparent editorial decisions that aggregate diverse opinions. ^90^ However, monitoring open platforms and ensuring quality interactive discussions might require significant commitment in terms of time and resources from the scientific community.
Put in place a hybrid two-tier system where only impactful research is sent for peer review	This solution posits that not all submissions are ‘worth’ going through the prepublication peer review process. Instead, a hybrid model with a two-tier system is applied ^86^: all submissions can be published and subjected to a post-publication peer review, but the prepublication peer review process needs only to evaluate manuscripts that are expected to have a significant impact on the field. ^86^ This is similar to some extent to desk-rejection practices where only submissions with high novelty and significance are sent for review. The main concern is of course how to make the initial evaluation about impact as objective as possible. This model, without safeguards, can end up biased toward top labs and renowned researchers. Moreover, it is unclear how citations from the unreviewed papers should be considered in the different research metrics.
Offer a non-selective review model to speed up the peer review process	Reviewers are often asked to make judgments about other aspects of the paper beyond its scientific merit, including, for instance, rating its novelty, significance, impact, originality, readability, and even whether or not the paper will be highly cited if accepted. The non-selective review model mainly focuses on papers’ scientific quality rather than their perceived importance and novelty. ^8^ Therefore, scientific peer reviewers only have to evaluate the methodological soundness of papers. It is also possible to go further along this model by asking reviewers only to review the methods and results sections. The other sections (including the authors’ own interpretations of the results in the Discussion section) can be open for discussion after publication.
Involve independent peer review platforms	An initial evaluation of the quality of papers is performed by other players. It is not unusual to hear scientific peer reviewers complaining about the fact that they are often asked to review papers of poor quality. They believe it is not their job to improve papers as this should be the task of the original authors. In this era of publishing as many and as fast as possible, some submissions poorly report the undertaken work, which might make the work of reviewers even harder. The idea is to involve other parties to help the authors improve their submissions before submitting them to a journal. This has been tested with the creation of independent peer review platforms, ^3^ helping researchers get their papers in good shape (for example, see platforms Rubriq and Editage). The authors pay the independent reviewers for their contribution to improving their papers. This model, however, might add extra costs to the preparation process of papers before submission to a journal.
Adopt a portable peer review across journals and publishers	This idea has been implemented by different initiatives (e.g. the Neuroscience Peer Review Consortium, the Review Commons). It is based on having a ‘portable’ peer review across publishers. ^91^ In this model, a paper goes through a journal-independent peer review, then the authors revise their paper accordingly and submit both their revised paper and the review reports to any journal taking part in the portable peer-review initiative. The revised submission can be moved among journals until a final editorial decision is made. ^37^ This model might help editors use reviewers time more efficiently but might delay the publication of papers (e.g. a paper might remain stuck in the process until it is selected by a suitable journal).
Create communities of researchers reviewing preprints	This model, promoted by “Peer Community in” (PCI), aims to organize the peer review process outside traditional journals. PCI is a non-profit scientific organization that creates communities of researchers to review and recommend preprints. It is based on an open-access system, a bottom-up process, and active scientific community engagement. ^92^ It has strong support from many research institutions and funding agencies. As in the case of many not-for-profit initiatives, scalability would be an issue without sufficient resources.
Merge journals into mega-journals with large editorial boards	A mega-journal (like PLOS One and Nature’s Scientific Reports) has a broad scope, accepting articles in almost all domains. It typically includes a large editorial board and a large pool of reviewers. Having relatively low rejection rates, mega-journals usually publish tens of thousands of articles yearly. They tend to have an efficient (fast turnaround time) peer review process that can handle large submission volumes. ^3^ They sometimes play a similar role as a cascade journal, publishing papers rejected by more selective top journals. ^93^ Because they rely on a large number of academic editors across multiple domains, they can efficiently involve peer reviewers. However, this mega-journal model might not serve the interests of all domains, as specialized journals are still needed.
Build an online reviewer registry accessible by all journals	The idea here is to build a large online registry of volunteer reviewers ^94^ who can be invited by any journal with access to the registry. This registry can be created in a collaborative way by different journals or publishers, including for instance, consortia of journals with overlapping scopes or domains. ^94^ Such registries can complement the reviewers databases that journals hold. The registry can include reviewers from diverse geographical locations, demographics and expertise. To sustain such initiatives, a cross-publisher partnership has to oversee the creation, maintenance, and update of the registry, with the mission to support it with the right resources.
Augment the review process with technology, including generative AI	Technology has already modernised the peer review process ^95^; e.g. automated detection of flawed methods or unethical practices like inappropriate statistical analysis, figure manipulation, or data fabrication. One can note the growing interest in developing powerful and versatile software and platforms to streamline the peer-review process (e.g. Scholastica). In the same way, it is expected that the adoption of generative AI will have significant ramifications on the peer review process in the near future. ^96^^,^^97^ Some of the applications of generative AI to peer review include identifying suitable reviewers, checking for any potential conflict of interest or risks of peer review manipulation, personalizing invitation emails and reminders, and assessing referee reports to assist editors in the decision process. Still, to minimize any risks of unethical practices, ^98^^,^^99^ an AI-enhanced peer review process needs human supervision. ^100^
You review as much as you publish	As the number of prolific (very productive) researchers is growing very fast, ^84^ this model requires active involvement from such frequent system users. Therefore, researchers who publish more should contribute more to the peer review process. ^101^ It is not unusual to see that many senior authors who publish a lot (and hence who are putting too much pressure on the system) are not involved at all in the peer review process. Some have even suggested that senior authors must provide evidence of their contribution to the peer review process as a requirement for considering their manuscripts for review. ^101^ This idea, albeit attractive, might not be feasible as it ignores the fact that senior authors are also very often involved in other academic review duties (doctoral theses, grant applications).
Offer incentives and rewards to reviewers	There is already a variety of rewards offered to reviewers to recognize their contribution to the peer review process. For instance, this includes prizes, discounts on publication fees, ORCID accreditation, free access to the publisher’s products (e.g. databases), Continuing Medical Education (CME) credits, vouchers for book purchases, reviewer certificates, reduced registration fees for conferences, recognition in databases, and many others. Moreover, active reviewers submitting quality reports can be added to the editorial board of the concerned journals. Despite these many incentives, one cannot deny that the number of available reviewers is still declining. Indeed, it is not well documented if these incentives are really useful for peer reviewers and how often they are claimed.
Link peer review involvement to the researcher’s academic benefits	Other incentives argue for linking promotion applications (e.g. for tenured positions) to how active the researcher is in the peer review process. ^102^ Likewise, other suggestions include earning continuing education credits upon completion of a review or waiving publication fees in the journal. ^103^ Others have even suggested offering co-authorship to reviewers, ^104^ in particular when the reviewers’ suggestions had significantly improved the original submission. However, this model might exclude those reviewers who are not ‎tenured academics.
Abandon the prepublication peer review model and adopt the post-publication peer-review (PPPR) model	The post-publication peer-review (PPPR) model ^105^^,^^106^ can ease the burden on journals with large submission volumes. It does not consider rejection after peer review. There is already rich literature about this model, ^89^ and it has been tested experimentally by different initiatives (e.g. F1000Research, ScienceOpen, The Winnower). Despite its many merits, this model has some limitations as it might make all papers available in the public domain, regardless of their original quality and impact. Without safeguards and an initial thorough check of paper quality, this model might end up mimicking the work of predatory journals that publish all submissions without (prepublication) peer review. Hence, PPPR must allow for continuous discussion and improvement of the quality of publications. ^105^ However, there has to be a limit on when a paper can remain open for discussion, as progress in science will lead to new experimental protocols and novel conceptual frameworks that will make ‘old’ publications look erroneous or obsolete.
Abandon the peer review process altogether	The (prepublication) peer review process should be abandoned as there is no strong evidence about its usefulness (e.g. see discussion in Ref. 14). The alternative is to gradually move toward expanding the role of preprint servers ^8^^,^^26^ or allowing universities and research institutions to manage their researchers’ publications. It is the researchers who create knowledge, and it is thus their responsibility to guarantee the accuracy and soundness of what they publish (accountability). It is also the responsibility of their institutions to disseminate their work in the format they deem fit.

However, these suggestions might not solve existing problems for five main reasons: (i) the number of submissions is projected to increase at much faster rates in this era of generative AI, (ii) the roles and duties of the academics include administration, writing grants, writing papers, writing patents, commercialization ventures, leaderships and directorships, editorships, teaching, supervision, committee memberships, consultancy, and outreach, leaving no time for reviewing papers (iii) the number of journals and publications in languages other than English is growing fast, promoted by current initiatives to improve inclusion in science dissemination, which makes it challenging to ensure a rigorous multilingual peer review process, (iv) researchers avoid peer-reviewing because they are increasingly unsatisfied ‎with for-profit publishers that reap exorbitant ‎profits while contributing negligibly towards academia and research, and (v) perhaps most importantly, researchers are likely to become less willing to spend time on reviewing AI-enhanced quick-to-write papers (e.g. when the time taken to review a paper becomes longer than the time taken to draft that paper with AI), leading to a growing reliance on generative AI by referees for peer review. I believe the latter is likely to be increasingly present in the debate about reforming the peer review process, which is why monetary incentives might be one potential means to alleviate the burden on the peer review process to some extent.

## Promoting professional reviewing as a career

Publishing research articles is still an expensive business, even in this digital era. ^44^^–^^46^ The global annual cost of peer review is estimated at around $1.5 billion, ^47^ corresponding to a net annual contribution of over 100 million hours by reviewers. ^48^ In the current science dissemination business, which is evaluated at billions of dollars, scientific peer reviewers still provide volunteer labour. Journals should thus develop methods to compensate reviewers for their time while maintaining the integrity of the peer-review process. ^47^^,^^49^ The system needs fair and sustainable mechanisms to pay referees. This would allow researchers to earn money by becoming full-time or part-time professional reviewers. Like the remunerated role played by professional language editors, researchers who enjoy evaluating research papers can be invited to dedicate more time to the peer review process for a fee. ^50^ For instance, a professional reviewer can be tasked with reviewing a given number of papers per week (e.g. 2 or 3 papers/week), which would help speed up the peer review process, in particular for journals with large submission volumes.

**Figure 1.**
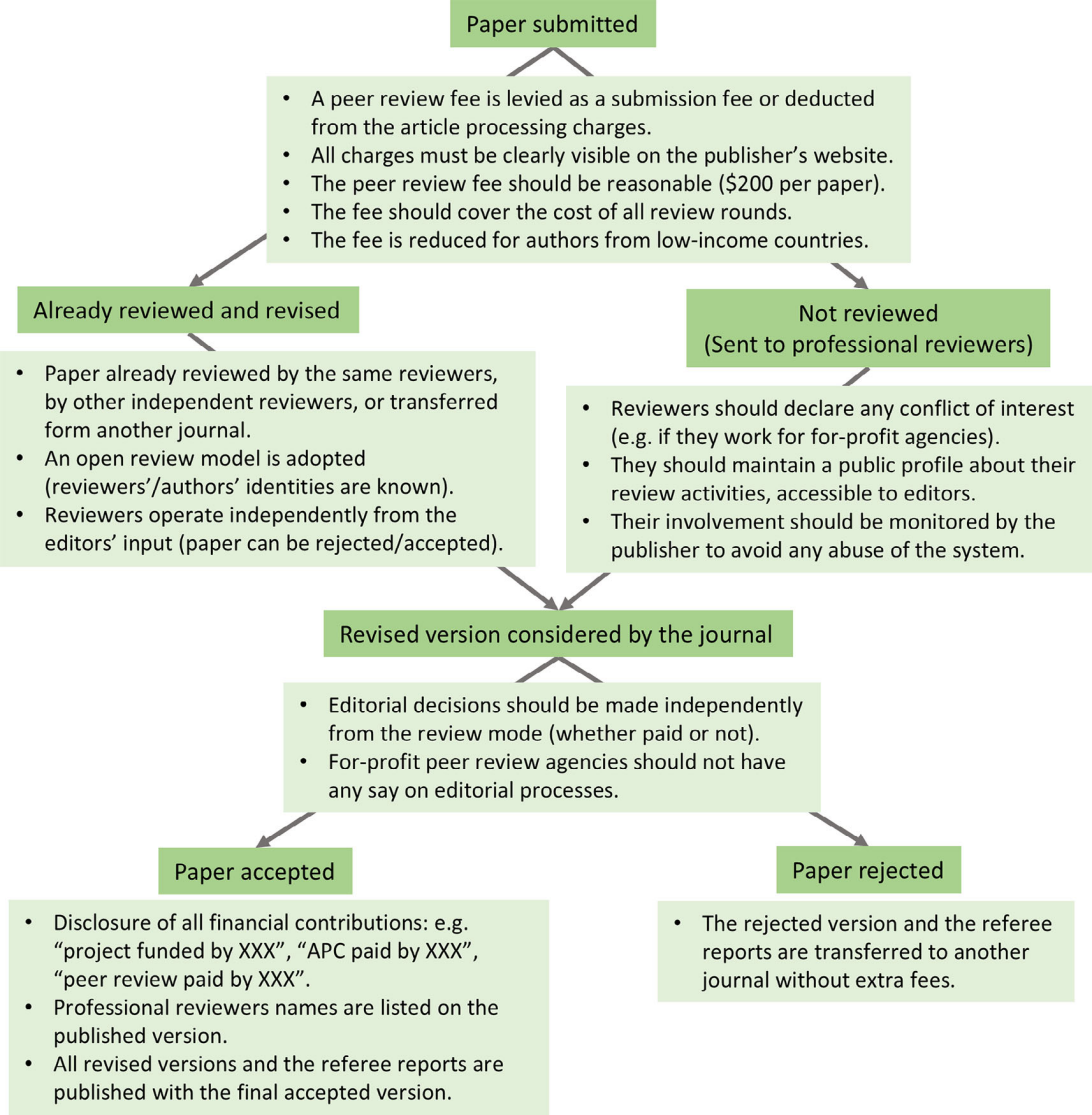
Professional reviewers can be an integral part of the peer review process. A typical publication process that involves professional reviewers. It shows the different steps (dark green boxes) and their implementation in a fair and transparent way (light green boxes). It is typically the case that review reports for manuscripts that get rejected are not published. To comply with open access publication standards and improve transparency on how public funds are used, review reports of rejected papers should be forwarded to the new journal handling the paper and published alongside the final accepted version. Transferring review reports to the new journal without extra fees should be managed by editors/journals to preserve the identity of anonymous reviewers. However, there is the risk that, unless all journals adopt an up-front reviewing fee, authors may submit to another journal without incorporating reviewers' suggestions or disclosing the existence of previous reviews.

We already have models where referees are paid for reviewing academic work, albeit at a smaller scale than article journals, as grant reviewers, PhD examiners, or academic promotion examiners. Reviewing articles is no exception and could thus be considered a remunerated task in academia. There is room for the emergence of two different but complementary professional reviewers. Reviewers can still be academicians, i.e. tenured staff and academics, working in universities and research institutions, where they can be offered the opportunity to supplement their salaries with additional honorariums for their active involvement in the peer review process. Another alternative concerns the possibility of outsourcing the peer review process by allowing the creation of independent providers. These providers, managed by for-profit companies, ^51^ can follow the same work model as companies that employ professional language editors in research dissemination. The job of a professional reviewer can be regulated by adopting new ethical and professional standards to safeguard the peer review process from any possible unethical practices that might occur when money is involved in the process ( Figure 1). Furthermore, peer-reviewing is also undertaken by non-tenured staff and academics with relevant ‎qualifications and expertise (i.e. postdocs, senior technical staff, research assistants, laboratory engineers and managers, ‎ and PhD-qualified persons who have quit academia). However, they generally cannot claim academic promotions for their voluntary participation in ‎the peer-review process. Thus, an inclusive peer review process must acknowledge their contributions by financially rewarding ‎them.

The proponent of this idea argues that peer review should be considered as a business transaction, ^52^ where a modest remuneration per paper of around $200, ^52^ or $450 for for-profit publishers, ^53^ can be offered to reviewers. This can motivate reviewers to be more actively involved in the peer review process and to offer fast and thorough review reports. ^54^^,^^55^ This can also be an incentive for retired scientists ^52^^,^^56^ to participate in peer review to gain an extra income that expands their retirement plans. Paying referees can increase the pool of available reviewers, including for instance researchers who cannot afford to work for free. ^56^ Many factors may interplay to determine the level of remuneration, ^48^^,^^57^^,^^58^ including reviewing for not-for-profit versus for-profit journals, scientific domains (e.g. research in medicine is more funded and expensive than research in art and humanities), the number of reviewers required per manuscript (e.g. two or more referees), the type of articles (e.g. reviewing a research article requires more hours than a commentary), and their length and complexity (e.g. reviewing a long review article or a full research article with complex data is more demanding than an opinion piece). Publishers and editors should estimate the level of remuneration according to the average hours typically spent by referees for each article type ^48^ and the average yearly number of manuscripts reviewed by each reviewer. ^39^^,^^58^ There is also the question of the type of financial compensation to referees: e.g. The ResearchHub Journal has recently started paying referees in cryptocurrency, ^59^ PeerJ journal offers tokens that can be redeemed against publication fees, and Biology Open journal started offering real money at the level of $300 per manuscript for freelance reviewers. ^60^ To guarantee fair and ethical payment schemes, active organizations and agencies should establish general guidelines about the payment of reviewers, including the Committee on Publication Ethics, the Declaration on Research Assessment, the Confederation of Open Access Repositories, and the European Association of Science Editors. Below, I discuss some options to sustain the payment of professional referees.

***Reviewers as consultants:*** Researchers are usually invited to budget for the support of a consultant or a technical expert when applying for grants. For instance, the consultant can provide expert opinion about an aspect of the research project that might be beyond the expertise of the researchers (e.g. a legal advisor, an industrial partner, a clinical professional, a computer system expert, a programmer, … etc). In the same way, a reviewer can be seen as a consultant whose role is to support the researchers in improving their papers in terms of readability, quality, and methodological soundness. Publications are typically included as deliverables in research grants, and therefore, the contribution of scientific peer reviewers to improve these deliverables can be budgeted as consultancy fees. Funding bodies may categorize this type of expense as publication fees. However, regardless of how paying reviewers is included in the budget, too much financial burden will be put on funding bodies as they already cover the excessive article-processing charges. Similar to some alternatives being sought for article-processing charges, ^61^ funding bodies should put in place guidelines to make the payment process of professional reviewers as fair and transparent as possible, particularly when for-profit agencies are managing the peer review process. For instance, many funding bodies already require that published work be made freely available, and thus similar requirements can also be made about the type of peer review (free or paid). If there is strong evidence that paying reviewers improves the quality of publications, then funding bodies can recommend researchers to publish in journals that pay referees. This ensures that funded research is being evaluated and scrutinized by the most rigorous review process that scholarly communication can offer (and afford).

***Levying a submission fee:*** As is the case in some journals, a submission fee can be collected for each submitted article, which can then be used to pay professional reviewers. For example, around 45 of the economics, finance, and accounting journals published by Elsevier charge a submission fee of around $50-$100. Likewise, some biology journals also levy submission fees. Their rationale is to ensure that only within-scope and high-quality papers are received. Submission fees also serve other purposes such as sponsoring conferences and workshops, the payment of language editing services, prizes/awards such as the annual best paper award, and even payments to top reviewers. To make the payment of professional reviewers sustainable, it might be suggested to levy a submission fee of $200 per submitted paper. For example, for a journal with a submission volume of 1,000 papers per year, and a desk-rejection rate of 50%, the collected submission fees (around $100,000) can help partially cover the costs of professional reviewers. To make this option attractive for authors, the submission fee should be deduced from the article-processing charges or the open access fee once the peer-reviewed paper is accepted. Consequently, this might mean that publishers should consider reducing their article processing charges when levying a submission fee. However, this might sound unsavory for for-profit publishers and hence the community needs to create an ecosystem that would make publishers active contributors to peer review, as discussed below. Likewise, having a submission fee that is “lost” upon rejection may deter authors from submitting to journals with up-front costs. This is why it would be more practical to integrate the submission fee into the overall publication charges applied by journals. Lastly, levying a submission fee might not be feasible for diamond open-access journals that do not charge authors and readers, and thus, they may still have to rely on voluntary referees.

***Paying referees in the context of economic disparity:*** One of the consequences of paying reviewers might be a better diversity in peer review. This is because paying referees might be very appealing for researchers from low- or middle-income countries. However, this monetary incentive can inadvertently yield over-involvement in the peer review process because a fee of $200 or more is sometimes comparable to a full salary in some low-income countries. Put another way, researchers from low-income countries can favour paid peer review over other academic duties, which might not be a tenable situation for their institutions. One way to avoid this race for more reviews, and hence more money, is to link the number of invitations for referees from low-income countries to the number of authors or readers from those low-income countries. For instance, if a journal has a readership of 20% from low-income countries, the number of invited referees from low-income countries can be capped at twice this percentage. This measure would also prevent for-profit publishers from taking advantage from the existing diversity by increasing reliance on referees from low-income countries and providing them with inadequate (low) compensation. Furthermore, all paid review tasks should be declared on the publisher’s website so that editors are well-informed about the level of involvement of different referees. This would help identify unusual review dynamics that need adjustments to ensure a fair and robust peer review.

***How about the publishers?*** The research community has voiced clear dissatisfaction about the ‘parasitic’ behaviour of some for-profit publishers in research dissemination. They are seen as the biggest winners of the current system despite not funding research and not paying the original content creators (the authors) and evaluators (the reviewers). ^62^ Recent examples of editors quitting for-profit journals provide an interesting illustration to appreciate the depth of that dissatisfaction (e.g. Refs. 63, 64). I believe that, if for-profit publishers do not engage with the research community more transparently and constructively, they will soon be made irrelevant to the whole process. Publishers must realize that they cannot continue operating along the same model. For instance, they can inject some of their profits back into the peer review process to support the remuneration of reviewers. However, it is unlikely that publishers would be willing to contribute financially to the peer review process unless they have control over the process through the creation of their professional review agencies. Still, I don’t think the research community can again trust for-profit publishers with the peer review process, which is understandable given the previous experience of the community with the open access model that has unfortunately been made so expensive and unaffordable by some for-profit publishers.

In this context, I feel that scholarly communication might foreseeably follow a radical model by completely bypassing for-profit publishers, i.e. a model that does not need the ‘middleman’. This implies that researchers and their universities will own the whole process. ^65^ In this model, universities and research institutions might choose to manage the whole publication process, for instance in the form of posting papers on open repositories, ^66^ while adopting an open post-publication peer review model. Universities can work as consortia to share the cost of publication through common or shared repositories, which can be further facilitated by the adoption of generative AI in the editorial process. ^67^^,^^68^ The other implication here is to allow researchers (or their institutions) to keep the copyright of their work instead of conceding it to publishers as in the current model.

***The influence of journal indexers:*** In addition to publishers, journal indexing bodies can play an important role in shaping the future of peer review (e.g. Web of Science, Scopus). Those journal indexers have the power to influence how journals are conducting peer review. Specifically, journal indexers assess the impact of various peer review systems on the quality of published papers. This assessment can lead to delisting journals that do not meet standards of scholarly communication, such as editorial integrity, peer review quality, and ethical publishing practices. The case of the journal eLife, removed by Web of Science from its Science Citation Index Expanded, offers an interesting example. The journal eLife, in addition to its embedded consultation model among peer reviewers, implemented a new (bold) publishing model called “Publish, Review, Curate”, in which manuscripts are first published as preprints and subsequent peer review reports and editorial assessments are published alongside the original preprints and revised articles. ^69^ However, Web of Science concluded that the new model conflicted with its quality assurance standards, leading to the journal not receiving an impact factor rating from Web of Science in 2025. ^70^ This also concerns many journals removed from the Scopus database for various reasons, including questionable peer-review processes.

Indexers are crucial in maintaining quality standards, such as delisting journals that manipulate the system or fail to offer rigorous peer reviews, as is often the case with many predatory journals. However, it is equally important for indexers to proceed cautiously in this regard, ensuring they do not hinder innovation in the peer review process by allowing sufficient time for new initiatives to prove their value, including the idea of paying reviewers. Overall, indexers should maintain an honest and pragmatic dialogue with all stakeholders to ensure that their objectives align with those of the scientific community, beyond financial or reputational considerations.

***Arguments against paying scientific peer reviewers:*** The opponents to paying reviewers mention the risks of proliferation of unethical practices (i.e. the system being corrupted by money) and unsustainable costs ^53^^,^^56^^,^^71^ as it can lead to increases in subscription fees and article processing charges. ^72^ Moreover, adding a monetary component to the peer review might lead to unethical practices to maximize the number of completed reviews, such as non-disclosure of conflict of interest, the tendency to review papers even outside one’s domain of expertise, and a fast turnaround time with no thorough evaluation of the reviewed papers. Therefore, journals should put some safeguards to ensure a high quality and rigorous peer review process, for instance, through the evaluation/rating by the editors of the quality of completed reviews before approving payment to the referees. However, such quality criteria are unfortunately still missing, given also the fact that that the expertise of reviewers and their personal style to provide feedback differs greatly. One potential alternative is to rely on structured peer reviews when implementing any payment schemes. Overall, perhaps the recent experiment at the journal Open Biology might provide a concrete example that peer review quality with paid reviewers can be guaranteed through a rigours editorial oversight and review. ^73^

It is also likely that a flat fee (e.g. $200 per manuscript) for all review rounds might create conflicting interests between reviewers and editors. Reviewers may be inclined to reject papers with extensive revisions, while editors prefer multiple revisions over rejections and resubmissions to avoid additional payments. Similarly, reviewers might not provide high quality feedback for additional review rounds when compensation is offered per manuscript. Journals may also invite fewer reviewers to reduce costs. Again, all these concerns underscore the need for robust safeguards to mitigate their negative impact on the overall quality of peer review.

Likewise, paying reviewers might yield a proliferation of for-profit peer review agencies with dubious ethical practices, like paper mills, that can generate fabricated review reports. ^72^ Some studies have argued that monetary rewards tend to decrease the quality of the peer review process ^74^ as well as the intrinsic motivation of researchers to contribute to the peer review process. ^75^ Paying reviewers might be only sustainable for journals with a reasonable submission volume. ^76^ For multidisciplinary journals with broad topics and large submission volumes, the peer review process can be enormously costly and thus diverse funding sources have to be sought. There is also the risk that paying referees will soon become the norm, making researchers no longer interested in reviewing for free. It could also lead to a multi-tier peer review system depending on how much a journal chooses to pay its reviewers, hence engendering an unequal competition among journals for the service of reviewers. This could hurt not-for-profit publishers that cannot afford an expensive peer review process. In that context, not-for-profit publishers must (i) diversify incentives on top of paying reviewers, (ii) nurture the intrinsic motivation of their readers to participate in peer review and serve their scientific community, ^77^ (iii) seek alternative means to be subsidized, ^78^ and (iv) foster strategic partnerships with all stakeholders to make not-for-profit journals the preferred model for open-access publicly-funded research. Overall, although one cannot rule out the risk of corruption in peer review by money, I trust that academia has built-in mechanisms to handle financial transactions fairly and objectively, as is the case when researchers are paid to evaluate grant proposals and academic theses. ^56^

***Other schemes of financial compensation:*** It might be worth mentioning other alternative models. For example, one proposal argues for offering monetary compensation not for the reviewers themselves but for their institutions ^79^ as they usually deal with the same publishers for purchasing subscriptions and other products. The rationale is that reviewers are already paid by their institutions, and hence, their involvement in the peer review process should benefit their institutions. However, this sometimes ignores the fact that researchers sometimes (often?) conduct their reviews outside work hours (e.g. evenings or weekends), and hence their contributions to peer review should not be assumed already covered by their salaries. Likewise, another recent proposal argues for monetary prizes not to individual referees but to their groups (departments, research units or labs). ^75^ That collected money, for instance, can support ongoing research projects, support early career members or the work of members from underrepresented minorities. ^75^

## Peer review cannot thrive in the current academic ecosystem

The above-mentioned solutions are assumed remedies to a not-fit-for-purpose peer review process. However, it is possible that the peer review process itself is adequate, and that the challenges encountered come from the environment within which peer review is conducted. Indeed, the culprit here is the current academic environment that promotes an unhealthy culture of “publish or perish”. To survive in this culture, researchers might fragment their results into multiple publications or even publish redundant papers based on similar data. ^37^^,^^80^ This culture of “publish or perish” is hurting academic research. ^81^ Therefore, instead of instigating calls to abandon or reform the peer review process, academia should invent and campaign for new models that depart from the “publish or perish” model. ^82^ As long as research quality is measured as a function of research productivity, there will be no form of peer review that can guarantee quality research while dealing with a deluge of papers that is now being inflated even more by the adoption of generative AI.

Is the ultimate goal of academia to produce prolific researchers who can publish several papers a week, ^83^ sometimes with questionable practices? ^84^ If, hypothetically, this goal is achieved by every researcher, who would review (and read) the published work? Evidence shows that many published papers remain uncited or end up having little impact on their respective fields, ^85^ but one cannot ignore the resources those papers took (time and effort) from reviewers and editors. It is as if the whole purpose of many substandard papers is to inflate some research metrics. Can academia promote a new model that puts a cap on how many papers an author can submit per year? This is essentially to invite researchers to think sensibly about the quality of their research and maybe only publish the best of their research that is likely to have an impact on their respective fields. Other work can still be shared as unreviewed preprints. ^86^ This framework ensures a manageable number of submissions, though it might entail a rethinking of current citation-based metrics of research impact and productivity.

## Conclusion

We can’t afford to ignore existing flaws in the current peer review system because the *status quo* will only favour the emergence of unethical practices. Here, I support the idea, albeit not new, of creating professional peer reviewers. Paying reviewers must be made fairly and transparently. Future work needs to assess if paying reviewers has a positive impact on the quality and rigor of peer review. Perhaps the recent “Fast & Fair peer review” experiment, run by the journal Biology Open where reviewers are paid for their work, offers some encouraging results about the positive impact of paying reviewers on the quality of peer review. ^60^ This experiment needs to be systematically tested in other journals and domains. Future work also needs to investigate alternative ways to ensure a sustainable and affordable remuneration of professional reviewers. The discussion should continue among all stakeholders to identify new solutions and innovations for efficient and affordable research dissemination in the modern era of generative AI. Empowering universities and research institutions to own the whole research dissemination system might soon be an inevitable scheme to consider. Finally, in the absence of effective and bold academic reforms, scholarly communication will continue relying on a costly and ineffective peer review system that will sooner or later be completely abandoned or replaced by something else.

## Contributions

This opinion article was conceived and written by MLS.

## Data Availability

No data are associated with this article.

## References

[1] Castillo M : Peer review: past, present, and future. *AJNR Am. J. Neuroradiol.* 2012;33(10):1833–1835. 10.3174/ajnr.A3025 22403775 PMC7964625

[2] Spier R : The history of the peer-review process. *Trends Biotechnol.* 2002;20(8):357–358. 10.1016/S0167-7799(02)01985-6 12127284

[3] Jubb M : Peer review: The current landscape and future trends. *Learn. Publ.* 2016;29(1):13–21. 10.1002/leap.1008

[4] Koshy K Fowler AJ Gundogan B : Peer review in scholarly publishing part A: why do it? *Int. J. Surg. Oncol.* 2018;3(2):e56. 10.1097/IJ9.0000000000000056

[5] Atkinson M : Regulation of Science by “Peer Review”. *Stud. Hist. Phil. Sci.* 1994;25:147–158. 10.1016/0039-3681(94)90025-6

[6] Baxt WG Waeckerle JF Berlin JA : Who reviews the reviewers? Feasibility of using a fictitious manuscript to evaluate peer reviewer performance. *Ann. Emerg. Med.* 1998;32(3 Pt 1):310–317. 10.1016/S0196-0644(98)70006-X 9737492

[7] Smith R : Peer review: a flawed process at the heart of science and journals. *J. R. Soc. Med.* 2006;99(4):178–182. 10.1177/014107680609900414 16574968 PMC1420798

[8] Walker R Rocha da Silva P : Emerging trends in peer review-a survey. *Front. Neurosci.* 2015;9:169. 26074753 10.3389/fnins.2015.00169 PMC4444765

[9] Haffar S Bazerbachi F Murad MH : Peer Review Bias: A Critical Review. *Mayo Clin. Proc.* 2019;94(4):670–676. 10.1016/j.mayocp.2018.09.004 30797567

[10] Rooyen S van : A critical examination of the peer review process. *Learn. Publ.* 1998;11(3):185–191. 10.1087/09531519850146355

[11] Williamson A : What will happen to peer review? *Learn. Publ.* 2003;16(1):15–20. 10.1087/095315103320995041

[12] Jefferson T Wager E Davidoff F : Measuring the quality of editorial peer review. *JAMA.* 2002;287(21):2786–2790. 10.1001/jama.287.21.2786 12038912

[13] Wager E Jefferson T : Shortcomings of peer review in biomedical journals. *Learn. Publ.* 2001;14(4):257–263. 10.1087/095315101753141356

[14] Heesen R Bright LK : Is Peer Review a Good Idea? *Br. J. Philos. Sci.* 2021;72(3):635–663. 10.1093/bjps/axz029

[15] Jefferson T Alderson P Wager E : Effects of editorial peer review: a systematic review. *JAMA.* 2002;287(21):2784–2786. 10.1001/jama.287.21.2784 12038911

[16] Relman AS : Peer review in scientific journals--what good is it? *West. J. Med.* 1990;153(5):520–522. 2260288 PMC1002603

[17] Chloros GD Giannoudis VP Giannoudis PV : Peer-reviewing in Surgical Journals: Revolutionize or Perish? *Ann. Surg.* 2022;275(1):e82–e90. 10.1097/SLA.0000000000004756 33630457

[18] Kelly J Sadeghieh T Adeli K : Peer Review in Scientific Publications: Benefits, Critiques, & A Survival Guide. *EJIFCC.* 2014;25(3):227–243. 27683470 PMC4975196

[19] Waltman L Kaltenbrunner W Pinfield S : How to improve scientific peer review: Four schools of thought. *Learn. Publ.* 2023;36(3):334–347. 10.1002/leap.1544 38504796 PMC10946616

[20] Jinha AE : Article 50 million: an estimate of the number of scholarly articles in existence. *Learn. Publ.* 2010;23(3):258–263. 10.1087/20100308

[21] Tennant JP : The state of the art in peer review. *FEMS Microbiol. Lett.* 2018;365(19). 10.1093/femsle/fny204 30137294 PMC6140953

[22] Larsen PO Ins M von : The rate of growth in scientific publication and the decline in coverage provided by Science Citation Index. *Scientometrics.* 2010;84(3):575–603. 10.1007/s11192-010-0202-z 20700371 PMC2909426

[23] Bornmann L Haunschild R Mutz R : Growth rates of modern science: a latent piecewise growth curve approach to model publication numbers from established and new literature databases. *Humanit. Soc. Sci. Commun.* 2021;8:224. 10.1057/s41599-021-00903-w

[24] Meyer HS Durning SJ Sklar DP : Making the First Cut: An Analysis of Academic Medicine Editors’ Reasons for Not Sending Manuscripts Out for External Peer Review. *Acad. Med.* 2018;93(3):464–470. 10.1097/ACM.0000000000001860 28767495

[25] Seghier ML : Demystifying desk rejection: A call to action for our authors. *Int. J. Imaging Syst. Technol.* 2022;32(3):701–703. 10.1002/ima.22733

[26] Gogotsi Y : Pay to publish? Open access publishing from the viewpoint of a scientist and editor. *Graphene 2D Mater.* 2023;8:1–3. 10.1007/s41127-023-00057-3

[27] Camargo LM Smirnov M Maldonado IL : The prey’s perspective on the rise of predatory publishing. *EXCLI J.* 2023;22:904–906. 10.17179/excli2023-6392 37780943 PMC10539542

[28] Grudniewicz A Moher D Cobey KD : Predatory journals: no definition, no defence. *Nature.* 2019;576(7786):210–212. 10.1038/d41586-019-03759-y 31827288

[29] Mertkan S Onurkan G Nilgun Suphi G : Profile of authors publishing in ‘predatory’ journals and causal factors behind their decision: A systematic review. *Res. Eval.* 2021;30(4):470–483. 10.1093/reseval/rvab032

[30] Rivera H : Fake Peer Review and Inappropriate Authorship Are Real Evils. *J. Korean Med. Sci.* 2018 Dec;34(2): e6. 10.3346/jkms.2019.34.e6 30636943 PMC6327091

[31] Rivera H : Teixeira da Silva JA: Retractions, Fake Peer Reviews, and Paper Mills. *J. Korean Med. Sci.* 2021;36(24): e165. 10.3346/jkms.2021.36.e165 34155837 PMC8216989

[32] McIntosh LD Hudson Vitale C : Safeguarding scientific integrity: A case study in examining manipulation in the peer review process. *Account. Res.* 2023:1–19. 10.1080/08989621.2023.2292043 38082492

[33] Kaltenbrunner W Pinfield S Waltman L : Innovating peer review, reconfiguring scholarly communication: an analytical overview of ongoing peer review innovation activities. *J. Doc.* 2022;78(7):429–449. 10.1108/JD-01-2022-0022

[34] Woods HB Brumberg J Kaltenbrunner W : An overview of innovations in the external peer review of journal manuscripts. *Wellcome Open Res.* 2022;7:82. 10.12688/wellcomeopenres.17715.1 36879926 PMC9984734

[35] Spier RE : Peer review and innovation. *Sci. Eng. Ethics.* 2002;8(1):99–108. discussion 109-12. 10.1007/s11948-002-0035-0 11840960

[36] Barroga E : Innovative Strategies for Peer Review. *J. Korean Med. Sci.* 2020;35(20):e138. 10.3346/jkms.2020.35.e138 32449322 PMC7246191

[37] Stahel PF Moore EE : Peer review for biomedical publications: we can improve the system. *BMC Med.* 2014;12:179. 10.1186/s12916-014-0179-1 25270270 PMC4177268

[38] Nguyen VM Haddaway NR Gutowsky LF : How long is too long in contemporary peer review? Perspectives from authors publishing in conservation biology journals. *PLoS One.* 2015;10(8): e0132557. 10.1371/journal.pone.0132557 26267491 PMC4533968

[39] Fernandez-Llimos F Salgado TM Tonin FS : How many manuscripts should I peer review per year? *Pharm. Pract (Granada).* 2020a;18(1):1804. 10.18549/PharmPract.2020.1.1804 32161628 PMC7055491

[40] Zupanc GKH : “It is becoming increasingly difficult to find reviewers”—myths and facts about peer review. *J. Comp. Physiol. A.* 2023;210:1–5. 10.1007/s00359-023-01642-w PMC10266957 37318565

[41] Ben Messaoud K Schroter S Richards M : Analysis of peer reviewers’ response to invitations by gender and geographical region: cohort study of manuscripts reviewed at 21 biomedical journals before and during covid-19 pandemic. *BMJ.* 2023;381:e075719. 10.1136/bmj-2023-075719 37311585 PMC10471900

[42] Ellwanger JH Chies JAB : We need to talk about peer-review-Experienced reviewers are not endangered species, but they need motivation. *J. Clin. Epidemiol.* 2020;125:201–205. 10.1016/j.jclinepi.2020.02.001 32061827

[43] Künzli N Berger A Czabanowska K : «I Do Not Have Time»—Is This the End of Peer Review in Public Health Sciences? *Public Health Rev.* 2022;43:1605407. 10.3389/phrs.2022.1605407 36467128 PMC9716458

[44] Grossmann A Brembs B : Current market rates for scholarly publishing services [version 2; peer review: 2 approved]. *F1000Res.* 2021;10:20. 10.12688/f1000research.27468.1 34316354 PMC8276192

[45] Smart P : Peer review: An expensive business. *Learn. Publ.* 2016;29:3–4. 10.1002/leap.1012

[46] Donovan B : The truth about peer review. *Learn. Publ.* 1998;11(3):179–184. 10.1087/09531519850146346

[47] LeBlanc AG Barnes JD Saunders TJ : Scientific sinkhole: estimating the cost of peer review based on survey data with snowball sampling. *Res. Integr. Peer Rev.* 2023;8(1):3. 10.1186/s41073-023-00128-2 37088838 PMC10122980

[48] Aczel B Szaszi B Holcombe AO : A billion-dollar donation: estimating the cost of researchers’ time spent on peer review. *Res. Integr. Peer Rev.* 2021;6(1):14. 10.1186/s41073-021-00118-2 34776003 PMC8591820

[49] Gasparyan AY Gerasimov AN Voronov AA : Rewarding peer reviewers: maintaining the integrity of science communication. *J. Korean Med. Sci.* 2015;30(4):360–364. 10.3346/jkms.2015.30.4.360 25829801 PMC4366954

[50] Ott S Hebenstreit D : Supply and demand: Apply market forces to peer review. *Nature.* 2014;506(7488):295. 10.1038/506295b 24553233

[51] Van Noorden R : Company offers portable peer review. *Nature.* 2013;494(7436):161. 10.1038/494161a 23407520

[52] Diamandis EP : Publishing costs: Peer review as a business transaction. *Nature.* 2015;517(7533):145. 10.1038/517145a 25567274

[53] Brainard J : The $450 question: Should journals pay peer reviewers? *Science.* 2021. 10.1126/science.abh3175

[54] Hamermesh DS : Facts and Myths about Refereeing. *J. Econ. Perspect.* 1994;8(1):153–163. 10.1257/jep.8.1.153

[55] Thompson GD Aradhyula SV Frisvold G : Does Paying Referees Expedite Reviews?: Results of a Natural Experiment. *South. Econ. J.* 2010;76(3):678–692. 10.4284/sej.2010.76.3.678

[56] Cheah PY Piasecki J : Should peer reviewers be paid to review academic papers? *Lancet.* 2022;399(10335):1601. 10.1016/S0140-6736(21)02804-X 35461548

[57] Huisman J Smits J : Duration and quality of the peer review process: the author’s perspective. *Scientometrics.* 2017;113:633–650. 10.1007/s11192-017-2310-5 29056794 PMC5629227

[58] Yankauer A : Who are the peer reviewers and how much do they review? *JAMA.* 1990;263(10):1338–1340. 10.1001/jama.1990.03440100042005 2304210

[59] Else H : ‘Getting paid to review is Justice’: Journal pays peer reviewers in cryptocurrency. *Nature.* 2024. (Accessed: 20 June 2025). 10.1038/d41586-024-04027-4 Reference Source 39663443

[60] Gorelick DA Clark A : Fast & Fair peer review: a pilot study demonstrating feasibility of rapid, high-quality peer review in a biology journal.bioRxiv 2025.03.18.644032.2025. 10.1101/2025.03.18.644032

[61] Sanderson K : Who should pay for open-access publishing? APC alternatives emerge. *Nature.* 2023;623(7987):472–473. 10.1038/d41586-023-03506-4 37964063

[62] Walter P Mullins D : From symbiont to parasite: the evolution of for-profit science publishing. *Mol. Biol. Cell.* 2019;30(20):2537–2542. 10.1091/mbc.E19-03-0147 31539315 PMC6740196

[63] Sanderson K : Editors quit top neuroscience journal to protest against open-access charges. *Nature.* 2023;616(7958):641. 10.1038/d41586-023-01391-5 37085706

[64] Singh Chawla D : Open-access row prompts editorial board of Elsevier journal to resign. *Nature.* 2019. 10.1038/d41586-019-00135-8

[65] Roh C : Owning the peer review process. *Coll. Res. Libr. News.* 2022;83(3). 10.5860/crln.83.3.100

[66] Torres L Hartley R : Repositories for academic products/outputs: Latin American and Chilean visions. *F1000Res.* 2019;8:1517. 10.12688/f1000research.19976.1 31700616 PMC6820816

[67] Conroy G : How ChatGPT and other AI tools could disrupt scientific publishing. *Nature.* 2023;622(7982):234–236. 10.1038/d41586-023-03144-w 37817033

[68] Kousha K Thelwall M : Artificial intelligence to support publishing and peer review: A summary and review. *Learned Publishing.* 2024;37(1):4–12. 10.1002/leap.1570

[69] Eisen MB Akhmanova A Behrens TE : Peer Review: Implementing a “publish, then review” model of publishing. *eLife.* 2020;9: e64910. 10.7554/eLife.64910 PMC7710353 33258772

[70] Open-access journal elife will lose its ‘impact factor’ over controversial publishing model | science | AAAS (no date a).(Accessed: 20 June 2025). Reference Source

[71] Gropp RE Glisson S Gallo S : Peer Review: A System under Stress. *Bioscience.* 2017;67(5):407–410. 10.1093/biosci/bix034

[72] Moustafa K : No to paid peer review. *Lancet.* 2022;400(10347):160. 10.1016/S0140-6736(22)01057-1 35843244

[73] Daniel AG Alejandra C : Fast & Fair peer review: a bold experiment in scientific publishing. *Biol. Open.* 15 March 2025;14(3): bio061982. 10.1242/bio.061982 PMC11972072 40129104

[74] Squazzoni F Bravo G Takacs K : Does incentive provision increase the quality of peer review? An experimental study. *Res. Policy.* 2013;42(1):287–294. 10.1016/j.respol.2012.04.014

[75] Bonaccorsi A : Towards Peer Review As a Group Engagement. *JLIS. It.* 2022;14(1):46–59. 10.36253/jlis.it-511

[76] Chang J-J Lai C-C : Is It Worthwhile to Pay Referees? *South. Econ. J.* 2001;68(2):457–463.

[77] Bonaccorsi A : Towards Peer Review As a Group Engagement. *JLIS. It.* 2022;14(1):46–59. 10.36253/jlis.it-511

[78] Johnson R : Not-for-profit scholarly publishing might not be cheaper – And that’s OK. *LSE.* 2024. Reference Source

[79] Copiello S : On the money value of peer review. *Scientometrics.* 2018;115:613–620. 10.1007/s11192-018-2664-3

[80] Beaufils P Karlsson J : Legitimate division of large datasets, salami slicing and dual publication. Where does a fraud begin? *Orthop. Traumatol. Surg. Res.* 2013;99(2):121–122. 10.1016/j.otsr.2013.01.001 23434431

[81] Rawat S Meena S : Publish or perish: Where are we heading? *J. Res. Med. Sci.* 2014;19(2):87–89. 24778659 PMC3999612

[82] Yeo MA Renandya WA Tangkiengsirisin S : Re-envisioning Academic Publication: From “Publish or Perish” to “Publish and Flourish”. *RELC J.* 2022;53(1):266–275. 10.1177/0033688220979092

[83] Ioannidis JPA Klavans R Boyack KW : Thousands of scientists publish a paper every five days. *Nature.* 2018;561(7722):167–169. 10.1038/d41586-018-06185-8 30209384

[84] Ioannidis JPA Collins TA Baas J : Evolving patterns of extremely productive publishing behavior across science. 2023. Preprint. 10.1101/2023.11.23.568476v2

[85] Van Noorden R : The science that’s never been cited. *Nature.* 2017;552(7684):162–164. 10.1038/d41586-017-08404-0 29239363

[86] Arns M : Open access is tiring out peer reviewers. *Nature.* 2014;515(7528):467. 10.1038/515467a 25428463

[87] Rooyen S van Godlee F Evans S : Effect of blinding and unmasking on the quality of peer review: a randomized trial. *JAMA.* 1998;280(3):234–237. 10.1001/jama.280.3.234 9676666

[88] Kennison R : Back to the future: (re) turning from peer review to peer engagement. *Learn. Publ.* 2016;29(1):69–71. 10.1002/leap.1001

[89] Ross-Hellauer T : What is open peer review? A systematic review [version 2; peer review: 4 approved]. *F1000Res.* 2017;6:588. 10.12688/f1000research.11369.1 28580134 PMC5437951

[90] Marcoci A Vercammen A Bush M : Reimagining peer review as an expert elicitation process. *BMC. Res. Notes.* 2022;15(1):127. 10.1186/s13104-022-06016-0 35382867 PMC8981826

[91] Bell GP Kvajo M : Tackling waste in publishing through portable peer review. *BMC Biol.* 2018;16(1):146. 10.1186/s12915-018-0619-z 30558673 PMC6297941

[92] Guillemaud T Facon B Bourguet D : Peer Community In: A free process for the recommendation of unpublished scientific papers based on peer review, in ELPUB 2019 23rd edition of the International Conference on Electronic Publishing.2019: Marseille, France.

[93] Spezi V Wakeling S Pinfield S : Open-Access mega-journals: The future of scholarly communication or academic dumping ground? A review. *J. Doc.* 2017;73:263–283. 10.1108/JD-06-2016-0082

[94] Ling F : Improving peer review: increasing reviewer participation. *Learn. Publ.* 2011;24:231–233. 10.1087/20110311

[95] Papatriantafyllou M : Peer Review - the future is here. *FEBS Lett.* 2017;591(18):2789–2792. 10.1002/1873-3468.12792 28805250

[96] Kousha K Thelwall M : Artificial intelligence to support publishing and peer review: A summary and review. *Learn. Publ.* 2024;37:4–12. 10.1002/leap.1570

[97] Heaven D : AI peer reviewers unleashed to ease publishing grind. *Nature.* 2018;563(7733):609–610. 10.1038/d41586-018-07245-9 30482927

[98] Singh Chawla D : Is ChatGPT corrupting peer review? Telltale words hint at AI use. *Nature.* 2024;628:483–484. 10.1038/d41586-024-01051-2 38600197

[99] Liang W Izzo Z Zhang Y : Monitoring AI-ModifiedContent at Scale: A Case Study on the Impact of ChatGPT on AI Conference Peer Reviews.arXiv.2024.

[100] Seghier ML : AI-powered peer review needs human supervision. *J. Inf. Commun. Ethics Soc.* 2025;23(1):104–116. 10.1108/JICES-09-2024-0132

[101] Graur D : Payback time for referee refusal. *Nature.* 2014;505:483. 10.1038/505483a 24451533

[102] Laxdal A Haugen T : Where are the carrots? A proposal to start crediting peer reviewers for their contribution to science. *Learn. Publ.* 2024;37:154–156. 10.1002/leap.1589

[103] Gorin MA : Combating reviewer fatigue with carrots. *BJUI Compass.* 2023;4(1):3–4. 10.1002/bco2.207 36569502 PMC9766864

[104] Kumar MN : The ‘peer reviewer as collaborator’ model for publishing. *Learn. Publ.* 2010;23(1):17–22. 10.1087/20100105

[105] Teixeira da Silva JA Dobranszki J : Problems with traditional science publishing and finding a wider niche for post-publication peer review. *Account. Res.* 2015;22(1):22–40. 10.1080/08989621.2014.899909 25275622

[106] Teixeira da Silva JA Al-Khatib A Dobranszki J : Fortifying the Corrective Nature of Post-publication Peer Review: Identifying Weaknesses, Use of Journal Clubs, and Rewarding Conscientious Behavior. *Sci. Eng. Ethics.* 2017;23(4):1213–1226. 10.1007/s11948-016-9854-2 27909954

