# Supplementary material for: Paying reviewers and regulating the number of papers may help fix the peer-review process
Source: F1000Res. 2025 Jun 24;13:439. Originally published 2024 May 2. [Version 4] doi: 10.12688/f1000research.148985.4 (PMC11221348; doi:10.12688/f1000research.148985.4)
Supplement: Peer review history

# Reviewer report: Reviewer response for version 4

**DOI:** 10.5256/f1000research.183781.r394344

**Published:** 2025-08-08

**Authors:** Robert Terry

**Affiliations:** 1 World Health Organization, Geneva, Switzerland

I’ve reviewed and approved the article. No further comments.

Is the topic of the opinion article discussed accurately in the context of the current literature?

Yes

Are arguments sufficiently supported by evidence from the published literature?

Yes

Are all factual statements correct and adequately supported by citations?

Yes

Are the conclusions drawn balanced and justified on the basis of the presented arguments?

Yes

Reviewer Expertise:

Research policy, open science, science communication,

I confirm that I have read this submission and believe that I have an appropriate level of expertise to confirm that it is of an acceptable scientific standard.

# Reviewer report: Reviewer response for version 4

**DOI:** 10.5256/f1000research.183781.r394339

**Published:** 2025-07-22

**Authors:** Toivo Glatz

**Affiliations:** 1 Institute of Public Health, Charité - Universitätsmedizin Berlin, Berlin, Germany

Thank you for the explanations and additions. I approve this revised version 4.

Is the topic of the opinion article discussed accurately in the context of the current literature?

Yes

Are arguments sufficiently supported by evidence from the published literature?

Yes

Are all factual statements correct and adequately supported by citations?

Yes

Are the conclusions drawn balanced and justified on the basis of the presented arguments?

Yes

Reviewer Expertise:

neuroscience of language, public health, epidemiology, peer review training

I confirm that I have read this submission and believe that I have an appropriate level of expertise to confirm that it is of an acceptable scientific standard.

# Reviewer report: Reviewer response for version 3

**DOI:** 10.5256/f1000research.170830.r359646

**Published:** 2025-03-06

**Authors:** Toivo Glatz

**Affiliations:** 1 Institute of Public Health, Charité - Universitätsmedizin Berlin, Berlin, Germany

Thank you for the opportunity to review the manuscript by Seghier, which is an opinion article on whether paying peer reviewers may help fix the scientific peer review process. The article provides a brief summary of some issues of scientific peer review and argues that there are not enough incentives to keep peer reviewers interested and motivated in agreeing to review manuscripts. Table 1 synthesizes the literature on proposed solutions for improving the peer review process and Figure 1 shows a publication system that includes the involvement of professional peer reviewers who should receive a fee of $200 for each reviewed paper to cover all rounds of review. The manuscript cites a wealth of references and gives a good overview and fair reflection of the current discussions surrounding scientific peer review.

 While I am in principle sympathetic towards the idea of paying peer reviewers, and much appreciate the work that went into synthesizing Table 1, I think the author’s suggestions fall a bit short of problems that can be expected as a consequence of following their suggestions. Being an opinion article, I do not expect the author to align their opinion with mine, but I hope that their arguments can be strengthened by considering some of my points below. I divide my feedback into more formal suggestions (comments 1-2) which should be implemented and more conceptual feedback (comments 3-9) formulating some of my own hypotheses which may or may not lead to changes in the manuscript.

  

1. Title and abstract use the term “paying reviewers”. Given that there are many different kinds of review(er)s, I would suggest using “scientific peer review(ers)” where possible.
2. Monetary units ($) lack a country (presumably US dollars?) and the dollar sign is inconsistently used after (200$) and before ($450) the number.
3. Payments should also depend on peer review quality: It is not uncommon that even at top journals reviewers submit reports that only contain a few words such as “accept as is”, or “seems ok”; giving no indication at all whether the manuscript was even read. Paying a flat fee to everybody who contributes to the process is therefore not ok. Individuals who want to become professional paid reviewers should have undergone some kind of training and their submitted reviews should fulfil some minimum quality criteria in order to qualify for receiving a fee. To my knowledge, such quality criteria are unfortunately still missing and calling for “journals should put some safeguards” is not sufficient given that journals are running a for-profit business. The research community should develop and agree on such safeguards instead. Given that the expertise of reviewers and their personal style to provide feedback differs greatly, it might also be best to put an emphasis on using structured peer reviews when implementing any payment scheme.
4. A flat fee covering all rounds of reviews creates opposing/competing incentives between reviewers and editors/journals: reviewers being invited to provide feedback on a paper that requires a lot of changes where many rounds of revisions can be anticipated might be more likely to suggest rejection and present shortcomings as crucial, whereas editors/journals have no interest in choosing “rejection with an option to resubmit” because this would mean they need to pay reviewers a second time, incentivising multiple rounds of revisions. Journals also have an incentive to invite fewer reviewers to cut down on costs.
5. Submission fees at some journals would encourage authors to submit to journals that do not have them: publicly funded research institutions are seeing budget cuts around the world, together with ever increasing publication costs, researchers (at least in Germany) are now incentivized to submit to journals for which an institutional agreement exists (instead of the ones that would be a good fit for a manuscript). I anticipate that having a submission cost which is “lost” in case of rejection will be extremely difficult to justify and authors will likely submit to journals that do not have up-front costs. The described submission fee of $200 would also not be able to cover the suggested $200-$450 per review report.
6. Interoperability of journals/publishers is required: Figure 1 shows the idea of transferring peer review reports to another journal without extra fees. While this would be the ideal situation, this could already be possible now but it does not work very well in practice. Given that this might include confidential information (such as the identity of anonymous reviewers and their conflict of interests) the communication cannot happen via the authors, but needs to go directly from one journal to another - given the fragmented manuscript submission systems this will be difficult and costly to implement. As long as not all journals implement an up-front reviewing fee, there is also nothing stopping authors from submitting at another journal without implementing any of the suggestions made by reviewers or even revealing that previous reviews exist.
7. All publicly funded peer reviews need to be openly available: even if journals employ an open peer-review process, this means that only review reports of manuscripts that get actually published are visible and part of the public record. Review reports for manuscripts that get rejected never see the light of day. In line with open access publication requirements, being transparent about what public money is used for, review reports of such rejected papers should also be public - which links to the previous point (6.) of moving reviews around. The only way I currently see to implement this, would be to preprint all manuscripts and only conduct open post-publication review, which seems unlikely to happen at large scale.
8. The author states that they have trust in built-in mechanisms in academia to prevent corruption of peer review by money. While I second that, it seems that these mechanisms are usually acting very slowly and only in response to issues after the situation has deteriorated over extended periods of time. A point worth mentioning might be that we have all these issues surrounding peer review because research institutions like universities decided to outsource quality control to commercial companies in the first place (e.g., by making publications in peer reviewed journals a requirement for a PhD and by using impact factors and citation rates as indicators for reward systems and promotions). Regaining some control over this process by providing protected time for peer reviews as part of contracts, having goals to achieve for contract extensions or tenure and keeping track of quality (see point 3 above) and quantity of reviews for each researcher instead of putting this once again in the hands of companies with commercial interests would be highly desirable.
9. The role of AI in alleviating some of the pressure on peer reviewers: the author lists generative AI as a problem for peer reviews as manuscripts might be produced faster than they can be reviewed. AI as a potential solution is only mentioned in passing and the author could elaborate for which tasks AI might help to expedite peer review (e.g., comments on adhering to reporting checklists, formatting or language) so that human reviewers can focus efforts on conceptual feedback.

Is the topic of the opinion article discussed accurately in the context of the current literature?

Yes

Are arguments sufficiently supported by evidence from the published literature?

Yes

Are all factual statements correct and adequately supported by citations?

Yes

Are the conclusions drawn balanced and justified on the basis of the presented arguments?

Yes

Reviewer Expertise:

neuroscience of language, public health, epidemiology, peer review training

I confirm that I have read this submission and believe that I have an appropriate level of expertise to confirm that it is of an acceptable scientific standard, however I have significant reservations, as outlined above.

# Response

**Published:** 2025-06-17

**Authors:** Mohamed Seghier

**Affiliations:** Khalifa University, United Arab Emirates

**I would like to thank the reviewer for their useful and constructive comments, I agree with all the comments and suggestions and revised the manuscript accordingly.**

 Point #1: Thank you for this suggestion. I have mentioned this in the abstract as well as throughout the manuscript.

 Point #2: Sorry about that. I have used the sign ‘$’ consistently before the number.

 Point #3: I fully agree with the reviewer here. Indeed, the system can be abused and that’s why I have mentioned some these risks. I have also added the following statement:

 “Moreover, adding a monetary component to the peer review might lead to unethical practices to maximize the number of completed reviews, such as non-disclosure of conflict of interest, the tendency to review papers even outside one’s domain of expertise, and a fast turnaround time with no thorough evaluation of the reviewed papers. Therefore, journals should put some safeguards to ensure a high quality and rigorous peer review process, for instance, through the evaluation/rating by the editors of the quality of completed reviews before approving payment to the referees. However, such quality criteria are unfortunately still missing, given also the fact that that the expertise of reviewers and their personal style to provide feedback differs greatly. One potential alternative is to rely on structured peer reviews when implementing any payment schemes. Overall, perhaps the recent experiment at the journal Open Biology might provide a concrete example that peer review quality with paid reviewers can be guaranteed through a rigours editorial oversight and review.”

 Point #4: These are important issues, and I don’t think I have an answer to all of them. They are now acknowledged in the manuscript as following: “It is also likely that a flat fee (e.g. $200 per manuscript) for all review rounds might create conflicting interests between reviewers and editors. Reviewers may be inclined to reject papers with extensive revisions, while editors prefer multiple revisions over rejections and resubmissions to avoid additional payments. Similarly, reviewers might not provide high quality feedback for additional review rounds when compensation is offered per manuscript. Journals may also invite fewer reviewers to reduce costs. Again, all these concerns underscore the need for robust safeguards to mitigate their negative impact on the overall quality of peer review.”

 Point #5: I agree here with the reviewer. The statement added is: " Likewise, having a submission fee that is “lost” upon rejection may deter authors from submitting to journals with up-front costs. This is why it would be more practical to integrate the submission fee into the overall publication charges applied by journals."

 Point #6: This is a very valid concern. I have added the following brief statement to Figure 1’s legend: “Transferring review reports to the new journal without extra fees should be managed by editors/journals to preserve the identity of anonymous reviewers. However, there is the risk that, unless all journals adopt an up-front reviewing fee, authors may submit to another journal without incorporating reviewers' suggestions or disclosing the existence of previous reviews.”

 Point #7: To acknowledge this matter, I have added the following statement to Figure 1’s legend: “It is typically the case that review reports for manuscripts that get rejected are not published. To comply with open access publication standards and improve transparency on how public funds are used, review reports of rejected papers should be forwarded to the new journal handling the paper and published alongside the final accepted version.”.

 Point #8: I fully agree with this comment. In fact, that is exactly what I tried to say in the second paragraphs of Section “How about the publishers?” and Section “Peer review cannot thrive in the current academic ecosystem”.

 Point #9: I have added new details to Table 1 about the role(s) of generative AI in this debate: “In the same way, it is expected that the adoption of generative AI will have significant ramifications on the peer review process in the near future. Some of the applications of generative AI to peer review include identifying suitable reviewers, checking for any potential conflict of interest or risks of peer review manipulation, personalizing invitation emails and reminders, and assessing referee reports to assist editors in the decision process. Still, to minimize any risks of unethical practices, an AI-enhanced peer review process needs human supervision”.

# Reviewer report: Reviewer response for version 3

**DOI:** 10.5256/f1000research.170830.r361817

**Published:** 2025-01-30

**Authors:** Robert Terry

**Affiliations:** 1 World Health Organization, Geneva, Switzerland

I note I am the 4th reviewer for this article - which is ironic in itself considering the topic!  I found it to be a good overview of the subject, and as an Opinion piece it offered different perspectives with a balanced presentation of the evidence. If the author has the  capacity the one area I feel is missing is the role of indexing (e.g. PubMed, Web of Science) as an additional 'final step' in quality assurance.  I mention this as indexers are playing a role in judging the impact of different peer review systems on the perceived quality of the published paper.  An example is the Web of Science which has decided to partially delist the journal eLife from its database and removed it from SCIE. WoS cites the reason as the potential for eLife to publish studies with inadequate or incomplete science, due to its use of the publish first and peer review subsequently model (publish, review, curate). cOAlition S (of which I am a member) published an opinion piece on this decision claiming it is stifling innovation in the publishing and peer review process ^1^

 This decision also perpetuates the false premise that a paper has a final version of record rather than the more realistic situation that errors and updates can corrected and added post V1 publication and, with good version control, are more representative of the dynamic nature of science. I provide this reference for consideration and for inclusion as a minor revision. ( https://www.coalition-s.org/blog/how-the-web-of-science-takes-a-step-back/)

Is the topic of the opinion article discussed accurately in the context of the current literature?

Yes

Are arguments sufficiently supported by evidence from the published literature?

Yes

Are all factual statements correct and adequately supported by citations?

Yes

Are the conclusions drawn balanced and justified on the basis of the presented arguments?

Yes

Reviewer Expertise:

Research policy, open science, science communication,

I confirm that I have read this submission and believe that I have an appropriate level of expertise to confirm that it is of an acceptable scientific standard.

# Reviewer report: Reviewer response for version 3

**DOI:** 10.5256/f1000research.170830.r359645

**Published:** 2025-01-27

**Authors:** Benjamin Speich

**Affiliations:** 1 University of Basel, Basel, Switzerland

Thank you very much for providing me the opportunity to review the article “ *Paying reviewers and regulating the number of papers may help fix the peer-review process*” written by Mohamed L. Seghier.

 The article is already in excellent shape, and I largely agree with its content (i.e. that peer reviewers are overburdened and that new solutions are required such as providing useful incentives to peer reviewers).

 I have a few minor comments which can be implemented as the author sees best fit:

  

1. Many journals offer already some sort of incentives such as publication of an annual list of peer reviewers’ names thanking them for their input , offering a discount on article processing charges, ORCID accreditation, Continuing Medical Education (CME) credits, and reviewer certificates (upon request). However, as far as I know it is not well known if these incentives are really useful for peer reviewers and how often they are claimed (e.g. discount on article processing can only be claimed if the peer reviewer submits an article within the same year to the journals as a corresponding author).
2. I liked the rational argumentation from the author. Still, I believe some points can be phrased more explicit. Is it for example fair that a few large publishers have an immense profit margin (supposed to be larger than Apple) while peer reviewers and academic editors usually work for free for the journal?
3. The author is proposing some changes in the system. I think these changes should be accompanied by research on this topic (e.g. assessing if paying reviewers has a positive impact on quality of publication).

Is the topic of the opinion article discussed accurately in the context of the current literature?

Yes

Are arguments sufficiently supported by evidence from the published literature?

Yes

Are all factual statements correct and adequately supported by citations?

Yes

Are the conclusions drawn balanced and justified on the basis of the presented arguments?

Yes

Reviewer Expertise:

Epidemiology; clinical research; meta-research

I confirm that I have read this submission and believe that I have an appropriate level of expertise to confirm that it is of an acceptable scientific standard.

# Reviewer report: Reviewer response for version 2

**DOI:** 10.5256/f1000research.168408.r300640

**Published:** 2024-07-18

**Authors:** Farid Rahimi

**Affiliations:** 1 Australian National University, Canberra, Australian Capital Territory, Australia

I thank the author for their considerations of my suggestions and for revising the manuscript.

Is the topic of the opinion article discussed accurately in the context of the current literature?

Yes

Are arguments sufficiently supported by evidence from the published literature?

Partly

Are all factual statements correct and adequately supported by citations?

No

Are the conclusions drawn balanced and justified on the basis of the presented arguments?

Yes

Reviewer Expertise:

Cell biology, neurodegeneration, inflammation, COVID-19, academic publishing and peer review.

I confirm that I have read this submission and believe that I have an appropriate level of expertise to confirm that it is of an acceptable scientific standard.

# Reviewer report: Reviewer response for version 2

**DOI:** 10.5256/f1000research.168408.r300638

**Published:** 2024-07-10

**Authors:** Vinícius Medina Kern

**Affiliations:** 1 Universidade Federal de Santa Catarina, Florianópolis, State of Santa Catarina, Brazil

Upon reviewing the manuscript v2, I acknowledge that my initial assessment did not identify any major mandatory changes, thus rendering the paper acceptable in its fundamental structure and content. The author has provided an 'Amendments from Version 1' section, which indicates some responsiveness to the feedback received.

 However, I must note with concern that the 'Minor Comments' from my initial review have largely been disregarded. These comments, though seemingly minor, are crucial for enhancing the clarity and readability of the paper. For instance, the citation format currently resembles the APA style, which may mislead readers into believing that the paper by Waltman (2023) is a sole authorship, whereas it is not. This discrepancy needs to be addressed, either by adhering strictly to the APA style or clearly indicating the use of an alternative referencing standard.

 Moreover, there are other minor yet significant issues highlighted in my initial review that remain unresolved. Ignoring these minor corrections could disrupt the reading experience and potentially misinform the audience.

 In conclusion, while the manuscript is fundamentally sound and makes a valuable contribution to the field, I recommend approval with restrictions. The author should address the minor corrections outlined in my initial review to ensure the manuscript meets the high standards expected by our readership.

Is the topic of the opinion article discussed accurately in the context of the current literature?

Yes

Are arguments sufficiently supported by evidence from the published literature?

Yes

Are all factual statements correct and adequately supported by citations?

Yes

Are the conclusions drawn balanced and justified on the basis of the presented arguments?

Yes

Reviewer Expertise:

educational peer review, scholarly communication, research evaluation

I confirm that I have read this submission and believe that I have an appropriate level of expertise to confirm that it is of an acceptable scientific standard, however I have significant reservations, as outlined above.

# Response

**Published:** 2024-08-19

**Authors:** Mohamed Seghier

**Affiliations:** Khalifa University, United Arab Emirates

Thank you for all these valuable comments. The previous revision considered the previous two reviewers' comments, and it was submitted before receiving Reviewer #3’s comments. That’s why Version 2 did not address the most recent comments of Reviewer #3. All these comments have now been addressed in this new revised version.

# Reviewer report: Reviewer response for version 1

**DOI:** 10.5256/f1000research.163380.r282628

**Published:** 2024-07-02

**Authors:** Vinícius Medina Kern

**Affiliations:** 1 Universidade Federal de Santa Catarina, Florianópolis, State of Santa Catarina, Brazil

This opinion paper makes a well-argued case in favor of paying referees. The article acknowledges both the literature in support of and against the argument.

 Major Comments:

 The case is parsimoniously introduced in the section "No more motivation to review for free", where it states that "monetary incentives might be one potential means to alleviate the burden on the peer review process to some extent" and that "paying professional reviewers can be implemented in conjunction with any of the solutions below" (in Table 1). Table 1 is an interesting summary of various kinds of proposed solutions to improve peer review.

 "Peer review cannot thrive in the current academic ecosystem" is a fortunate name for the section before Conclusion. While I think it is enough for this paper, it only touches the tip of the iceberg. The literature on peer review is rich, but it is scarce in describing and explaining peer review as a functioning system. The current system appears to serve commercial publishers, who profit significantly from it, far better than it does authors, referees, and often under-compensated editors. The components, environment, and interconnections that underpin the system and drive its dynamics require further exploration. The Open Access movement has successfully promoted free readership, which seems good and logical given the public funding behind most research. However, it has not succeeded in reducing the costs exacerbated by the Serials Crisis, that motivated the movement.

 Furthermore, the peer review system has historically failed the research assessment culture by keeping reviews hidden from all but the editor, the author, and the referees (except for their own reviews). This has resulted in decades of systemic non-learning in paper assessment. The Open Science Movement now advocates for open reviews, a commendable initiative, though not always feasible for several reasons. The *lack of shared reviews among referees over the decades* remains a significant shortcoming. Making everything open is quite a radical change. This is clearly not a critique of this paper but highlights a broader systemic issue.

 Regarding potential hindrances to implementing reviewer payment, I suggest the author consider mentioning the fact that the proposed solution is hardly feasible for journals in Diamond Open Access - the ones that do not charge from author nor readers, mostly from poorer countries. They mostly rely on voluntary work, with some support from universities or government funding.

 Minor Comments:

 The correct citation for item 18 ("according to Waltman(2023)") seems to be "according to Waltman et al. (2023)."

 Some editing might improve readability. For instance, in "Those qualities, promoted by other schools of thoughts, are ...", my understanding halted so I could think if the school of thought adopted also promotes those qualities and if the other schools of thought are the three other ones described by Waltman et al. (2023). It would be clear to me and the disturbance would not occur if the segment was written as "Those qualities, also promoted by the other schools of thought, are ..."

 The article effectively uses the current literature, but I feel some statements lack a citation nonetheless. For instance, "... increase in instances of fraud where for example false contact information of potential referees with non-institutional emails are suggested or when the peer review process has been manipulated" could be backed by sources such as [1] or [2] (for instance).

 I think it would be appropriate to mention Publons (Table 1, p. 6) pointing out that it was bought by Clarivate and does not exist under that name, or, at least, to give a link (http://webofscience.help.clarivate.com/Content/publons.html). Future readers might be unable to find out what Publons was.

 In Figure 1, the blue and green boxes are difficult to distinguish. They appeared identical to me - until I read the figure label.

 There's are typos that I suppose either the author of the editorial team will correct, such as "no through evaluation of the reviewed papers" ("thorough") and "Is the ultimate goal of academia to produce ... sometimes with questionable practices." (missing interrogation mark).

Is the topic of the opinion article discussed accurately in the context of the current literature?

Yes

Are arguments sufficiently supported by evidence from the published literature?

Yes

Are all factual statements correct and adequately supported by citations?

Yes

Are the conclusions drawn balanced and justified on the basis of the presented arguments?

Yes

Reviewer Expertise:

educational peer review, scholarly communication, research evaluation

I confirm that I have read this submission and believe that I have an appropriate level of expertise to confirm that it is of an acceptable scientific standard.

## References

[1] : Retractions, Fake Peer Reviews, and Paper Mills. *J Korean Med Sci* .2021;36(24) : 10.3346/jkms.2021.36.e165 e165 10.3346/jkms.2021.36.e165 34155837 PMC8216989

[2] Paying reviewers and regulating the number of papers may help fix the peer-review process. *https://www.researchgate.net/publication/358423532_Exploratory_analysis_of_text_duplication_in_peer-review_reveals_peer-review_fraud_and_paper_mills/citation/download* .2022; 10.1007/s11192-022-04504-5 10.1007/s11192-022-04504-5 PMC1122134838962691

# Reviewer report: Reviewer response for version 1

**DOI:** 10.5256/f1000research.163380.r282627

**Published:** 2024-06-21

**Authors:** Farid Rahimi

**Affiliations:** 1 Australian National University, Canberra, Australian Capital Territory, Australia

Dr Seghier has written a comprehensive narrative opinion piece on the peer-review process as it is an integral part of the academic ecosystem. Dr Seghier has highlighted the fundamental issues that challenge(d) the efficacy and efficiency of the peer-review process contemporarily (or historically) and argues for establishing a transparent system whereby peer reviewers could be financially rewarded and recompensed. De Seghier argues that capping the number of paper submissions may also help reform the peer-review process.

 I have listed my comments that I hope will help improve the opinion piece or open up the perspective to discussion. The manuscript is written relatively well; however, summarising the text will benefit the readers.

 Major comments:

 I suggest adding the emergence of competing mega-journals or mega-publishers as another issue ‎that may affect the peer-review process (in the Abstract). This was mentioned in Table 1 and in reference 70.‎ I believe this is part of the problem.

 The last statement of the Abstract contradicts the argument for reform. Please revise “Instead of ‎reforming the peer-review process …” because the piece calls for reform.‎

 Change the somehow hypothetical, “It can exist …” to factual, “It exists …” or “Peer review is ‎performed in different ways.” ‎

 Please note that the description of the traditional roles of academics includes or represents the mainstream tenured academics. However, ‎peer-reviewing is undertaken by non-tenured staff and academics with relevant ‎qualifications, experience, and expertise. Reviewers in this category (for example, postdoctoral ‎scholars, senior technical officers, qualified research assistants, qualified laboratory managers, ‎or PhD-qualified persons who have quit academia, qualified academics who are active in postgraduate teaching) undertake peer-review merely to serve the ‎scientific community; however, they generally cannot claim academic promotions for their voluntary participation in ‎the peer-review process (because they do not have grants or tenured positions in a faculty). Thus, financially rewarding ‎them makes perfect sense for their service and inclusion.‎ Such an understanding acknowledges the inclusivity of the peer review process.

 Similarly, the suggestion of co-authorship and academic benefits and promotions is interesting as ‎presented in the Table 1. However, this excludes those reviewers who are not ‎tenured academics (as discussed above). ‎Please revise the paragraph 2 in page 7 of 13 (PDF file) to remove the assumption or assertion that all reviewers ‎are academicians. ‎

 Please note that the remuneration of $200 (in Table 1 and page 8 of 13) or $450 are ‎prescriptive. Some reviewing tasks demand many valuable hours if done diligently. Larger pieces of work and larger manuscripts with complex data and messages will require more time for thorough assessment. A mechanism ‎for determining this remuneration should be discussed instead of prescribing a fixed amount (many factors may interplay). ‎Determining the remuneration may not be simple as suggested or alluded by the author in the ‎context of low-income countries.‎ If this is left with the publishers, or some publishers take on this initiative or control, the publication charges and profit reaping will increase by many folds. What body controls this is unimaginable.

 Please revise the assumptions that the funding bodies cover the publication charges and how ‎they should work closely with the publishers to establish a fair means of paying the reviewers. ‎This is confusing to me because, as I understand, funding bodies provide the grants to the academics ‎or the researchers. The latter then need to budget for publication charges out of their granted ‎funds. I also understand that the funding bodies normally do not interact with the publishers that ‎publish a funded project that was conducted by the funded researchers or academics. Funding bodies also do not interfere with the research plans or outcomes. ‎

 Please note that Publons does not exist anymore. Publons was a commercial website that ‎provided a free service for academics to track, verify, and showcase their peer review and ‎editorial contributions to academic journals. It was launched in 2012 but was bought by Clarivate ‎Analytics in 2017. https://en.wikipedia.org/wiki/Publons.‎

 Minor comments:

 The title is wordy. Please consider rephrasing/summarizing, e.g., “Paying academic reviewers and regulating the number of submitted papers may help fix the peer-review process.” Or “Remunerating peer reviewers and restricting the number of submitted papers may help fix the peer-‎review process.‎”

 Please rewrite “It might be useful to contemplate what could be the alternative if no adequate and sustainable solutions are found.” to remove the dummy subject “it” and the hypothetical nature of the statement and clarify the message. For example, “Finding adequate and sustainable alternative solutions will be useful [or “is needed”].”

 Please revise and summarize the following sentence and juxtapose “include or including” with the roles or duties. For example, “the traditional job of academia is being transformed with extra roles that researchers are expected to assume, leaving no time for reviewing papers, including admin duties, seeking funds, leadership roles, academic advising, organizing outreach activities in the community, and working on translating their research into marketable solutions,” should be changed to “the roles and duties of the academics include administration, writing grants, writing papers, writing patents, commercialization ventures, leaderships and directorships, editorships, teaching, supervision, committee memberships, consultancy, and outreach, leaving no time for reviewing papers.”

 Please change “in other languages than English” to “in languages other than English”.‎

 Please summarize the following statement to remove repetitions: “researchers are increasingly ‎unsatisfied with for-profit publishers that have made exorbitant profits with negligible contribution to ‎research in general, resulting in many researchers not keen to review for for-profit journals”. For ‎example, “academics and authors avoid peer-reviewing because they are increasingly unsatisfied ‎with for-profit publishers that reap exorbitant ‎profits while contribute negligibly towards academia and research.” While mentioning this, some journals or publishers sponsor conferences or provide thesis or paper prizes and competitions. However, the proportion of this contribution could be small. I’m not sure if this has been studied systematically. ‎

 In Table 1, revise the following:

 “basically based on”

 “Reviewers time” to “Reviewers’ time”

 “Add some delays to the publication of papers” to “Delays the publication”

 “Reviewers’ databases”

 “to oversight” to “to oversee”

 “Augment the peer process” to “Augment the review process”

 “are not Involved” to “are not involved”

 Rephrase the subtitle, “Toward the promotion of Professional Reviewer as a career” to “Promoting professional reviewing as a career”. A review cannot be factually (logically) promoted as a career.

 Please rephrase sentences that use dummy subjects, “it is,” “there is,” or “there are” as suggested by the principles of plain English and for the sake of brevity.

 Please avoid personifications, for example, “… this paper reckons …” while the credit should go to the author.

 Avoid using the passive voice and nominalizations (using verbs as nouns requiring helper verbs). For example, “Many calls have been made to reform …”

 Avoid contractions in academic writing, e.g., “I’m.”

 Correct the minor spelling mistakes:

 ‘chose’ [choose in two places]

 ‘through evaluation’ [thorough evaluation]

 between journals [among journals]

 add a question mark after ‘Is the ultimate …’,

 ‘will soon or later’ [will sooner or later] …

 The paper will benefit from proofreading, editing, and summarization.

 Thank you for the opportunity to review your work.

Is the topic of the opinion article discussed accurately in the context of the current literature?

Yes

Are arguments sufficiently supported by evidence from the published literature?

Partly

Are all factual statements correct and adequately supported by citations?

No

Are the conclusions drawn balanced and justified on the basis of the presented arguments?

Yes

Reviewer Expertise:

Cell biology, neurodegeneration, inflammation, COVID-19, academic publishing and peer review.

I confirm that I have read this submission and believe that I have an appropriate level of expertise to confirm that it is of an acceptable scientific standard, however I have significant reservations, as outlined above.

# Response

**Published:** 2024-06-25

**Authors:** Mohamed Seghier

**Affiliations:** Khalifa University, United Arab Emirates

**Point-by-point responses:**

**I would like to thank both reviewers for their very helpful and constructive comments. All their concerns have been addressed in this resubmitted revision.**

**All changes are highlighted in the revised manuscript** **with track changes.**

**Below, is a detailed point-by-point rebuttal addressing each reviewer’s comment.**

**Reviewer #2:** Farid Rahimi, Australian National University, Canberra, Australian Capital Territory, Australia.

 Dr Seghier has written a comprehensive narrative opinion piece on the peer-review process as it is an integral part of the academic ecosystem. Dr Seghier has highlighted the fundamental issues that challenge(d) the efficacy and efficiency of the peer-review process contemporarily (or historically) and argues for establishing a transparent system whereby peer reviewers could be financially rewarded and recompensed. De Seghier argues that capping the number of paper submissions may also help reform the peer-review process. I have listed my comments that I hope will help improve the opinion piece or open up the perspective to discussion. The manuscript is written relatively well; however, summarising the text will benefit the readers.

**Thank you very much for your positivity and the very useful suggestions.**

**Reviewer #2, continued:**

 Major comments:

 I suggest adding the emergence of competing mega-journals or mega-publishers as another issue ‎that may affect the peer-review process (in the Abstract). This was mentioned in Table 1 and in reference 70.‎ I believe this is part of the problem.

**Response:**

**Thank you for this very interesting suggestion. I have briefly mentioned this issue in the new version of the Abstract: “ *the emergence of competing mega-journals*”.**

**Reviewer #2, continued:**

 The last statement of the Abstract contradicts the argument for reform. Please revise “Instead of ‎reforming the peer-review process …” because the piece calls for reform.‎

**Response:**

**I fully agree with the reviewer. Accordingly, I have deleted that statement from the Abstract.**

**Reviewer #2, continued:**

 Change the somehow hypothetical, “It can exist …” to factual, “It exists …” or “Peer review is ‎performed in different ways.” ‎

**Response:**

**Done! Thank you.**

**Reviewer #2, continued:**

 Please note that the description of the traditional roles of academics includes or represents the mainstream tenured academics. However, ‎peer-reviewing is undertaken by non-tenured staff and academics with relevant ‎qualifications, experience, and expertise. Reviewers in this category (for example, postdoctoral ‎scholars, senior technical officers, qualified research assistants, qualified laboratory managers, ‎or PhD-qualified persons who have quit academia, qualified academics who are active in postgraduate teaching) undertake peer-review merely to serve the ‎scientific community; however, they generally cannot claim academic promotions for their voluntary participation in ‎the peer-review process (because they do not have grants or tenured positions in a faculty). Thus, financially rewarding ‎them makes perfect sense for their service and inclusion.‎ Such an understanding acknowledges the inclusivity of the peer review process. Similarly, the suggestion of co-authorship and academic benefits and promotions is interesting as ‎presented in the Table 1. However, this excludes those reviewers who are not ‎tenured academics (as discussed above). ‎Please revise the paragraph 2 in page 7 of 13 (PDF file) to remove the assumption or assertion that all reviewers ‎are academicians. ‎

**Response:**

**Thank you for raising this important point. Accordingly, I have made the following changes:**

**To Table 1: I have added this brief statement: “ *However, this model might exclude those reviewers who are not*** ***‎*** ***tenured academics.*** **”.**

**To Page 6, the edited text now reads: “ *Furthermore, peer-reviewing is also undertaken by non-tenured staff and academics with relevant*** ***‎*** ***qualifications and expertise (i.e. postdocs, senior technical staff, research assistants, laboratory engineers and managers,*** ***‎*** ***and PhD-qualified persons who have quit academia). However, they generally cannot claim academic promotions for their voluntary participation in*** ***‎*** ***the peer-review process. Thus, an inclusive peer review process must acknowledge their contributions by financially rewarding*** ***‎*** ***them.*** **”**

**Reviewer #2, continued:**

 Please note that the remuneration of $200 (in Table 1 and page 8 of 13) or $450 are ‎prescriptive. Some reviewing tasks demand many valuable hours if done diligently. Larger pieces of work and larger manuscripts with complex data and messages will require more time for thorough assessment. A mechanism ‎for determining this remuneration should be discussed instead of prescribing a fixed amount (many factors may interplay). ‎Determining the remuneration may not be simple as suggested or alluded by the author in the ‎context of low-income countries.‎ If this is left with the publishers, or some publishers take on this initiative or control, the publication charges and profit reaping will increase by many folds. What body controls this is unimaginable.

**Response:**

**Thank you for raising this important point. The two figures ($200 and $450) were in fact suggested in prior work. I agree that many factors influence the level of remuneration, and I don’t think that a single mechanism can determine the exact remuneration for so many journals in very diverse domains. Having said that, I have added the following paragraph to Page 7:**

**“ *Many factors may interplay to determine the level of remuneration [45, 54, 55], including reviewing for not-for-profit versus for-profit journals, scientific domains (e.g. research in medicine is more funded and expensive than research in art and humanities), the number of reviewers required per manuscript (e.g. two or more referees), the type of articles (e.g. reviewing a research article requires more hours than a commentary), and their length and complexity (e.g. reviewing a long review article or a full research article with complex data is more demanding than an opinion piece). Publishers and editors should estimate the level of remuneration according to the average hours typically spent by referees for each article type [45] and the average yearly number of manuscripts reviewed by each reviewer [36, 55]. Other organizations and agencies can also establish general guidelines about the payment of reviewers, including the Committee on Publication Ethics, the Declaration on Research Assessment, the Confederation of Open Access Repositories, and the European Association of Science Editors.*”**

**Reviewer #2, continued:**

 Please revise the assumptions that the funding bodies cover the publication charges and how ‎they should work closely with the publishers to establish a fair means of paying the reviewers. ‎This is confusing to me because, as I understand, funding bodies provide the grants to the academics ‎or the researchers. The latter then need to budget for publication charges out of their granted ‎funds. I also understand that the funding bodies normally do not interact with the publishers that ‎publish a funded project that was conducted by the funded researchers or academics. Funding bodies also do not interfere with the research plans or outcomes. ‎

**Response:**

**Thank you for allowing me to clarify this statement. Accordingly, I have edited that paragraph as below (see Page 7):**

**“ *Publications are typically included as deliverables in research grants, and therefore the contribution of peer reviewers to improve these deliverables can be budgeted under the category of consultancy fees. Funding bodies may categorize this type of expense as publication fees. However, regardless of how paying reviewers is included in the budget, too much financial burden will be put on funding bodies as they are already covering the excessive article-processing charges. As alternatives are being sought for article-processing charges [56], funding bodies should put in place guidelines to make the payment process of professional reviewers as fair and transparent as possible, in particular when for-profit agencies are managing the peer review process. For instance, many funding bodies already require that published work must be made freely available, and thus similar requirements can also be made about the type of peer review (free or paid). If there is strong evidence that paying reviewers improves the quality of publications, then funding bodies can recommend researchers to publish in journals that pay referees. This ensures that funded research is being evaluated and scrutinized by the most rigorous review process that scholarly communication can offer (and afford).*”**

**Reviewer #2, continued:**

 Please note that Publons does not exist anymore. Publons was a commercial website that ‎provided a free service for academics to track, verify, and showcase their peer review and ‎editorial contributions to academic journals. It was launched in 2012 but was bought by Clarivate ‎Analytics in 2017. https://en.wikipedia.org/wiki/Publons.‎

**Response:**

**Although the brand name Publons ceased to exist, some of its functionalities are still available via Web of Science. To avoid any confusion, I have now deleted it from Table 1.**

**Reviewer #2, continued:**

 Minor comments:

 The title is wordy. Please consider rephrasing/summarizing, e.g., “Paying academic reviewers and regulating the number of submitted papers may help fix the peer-review process.” Or “Remunerating peer reviewers and restricting the number of submitted papers may help fix the peer-‎review process.‎”

**Response:**

**As suggested by the reviewer, I have now added the following new title: “ *Paying reviewers and regulating the number of papers may help fix the peer-review process*”.**

**Reviewer #2, continued:**

 Please rewrite “It might be useful to contemplate what could be the alternative if no adequate and sustainable solutions are found.” to remove the dummy subject “it” and the hypothetical nature of the statement and clarify the message. For example, “Finding adequate and sustainable alternative solutions will be useful [or “is needed”].”

**Response:**

**This statement has been rewritten as recommended by the reviewer; see Page 3.**

**Reviewer #2, continued:**

 Please revise and summarize the following sentence and juxtapose “include or including” with the roles or duties. For example, “the traditional job of academia is being transformed with extra roles that researchers are expected to assume, leaving no time for reviewing papers, including admin duties, seeking funds, leadership roles, academic advising, organizing outreach activities in the community, and working on translating their research into marketable solutions,” should be changed to “the roles and duties of the academics include administration, writing grants, writing papers, writing patents, commercialization ventures, leaderships and directorships, editorships, teaching, supervision, committee memberships, consultancy, and outreach, leaving no time for reviewing papers.”

**Response:**

**Thank you for this suggestion. I have added it to Page 5 of the new revised version.**

**Reviewer #2, continued:**

 Please change “in other languages than English” to “in languages other than English”.‎

**Response:**

**Done! Thank you.**

**Reviewer #2, continued:**

 Please summarize the following statement to remove repetitions: “researchers are increasingly ‎unsatisfied with for-profit publishers that have made exorbitant profits with negligible contribution to ‎research in general, resulting in many researchers not keen to review for for-profit journals”. For ‎example, “academics and authors avoid peer-reviewing because they are increasingly unsatisfied ‎with for-profit publishers that reap exorbitant ‎profits while contribute negligibly towards academia and research.” While mentioning this, some journals or publishers sponsor conferences or provide thesis or paper prizes and competitions. However, the proportion of this contribution could be small. I’m not sure if this has been studied systematically. ‎

**Response:**

**I have replaced that statement with the reviewer’s suggestion (see Page 5 of the new revised version).**

**Regarding your second point, I’m not aware of any systematic analysis of the contribution of for-profit publishers to scholarly communication. While they do sponsor some academic events, I believe this is a small fraction of what they get as profits (e.g. according to some estimations, Elsevier for instance has a 40% profit margin on its $2 Billion annual revenue). I did not mention this point in the revised version because it is extremely hard to get the exact figures when it comes to the money made or spent by publishers.**

**Reviewer #2, continued:**

 In Table 1, revise the following:

 “basically based on”

 “Reviewers time” to “Reviewers’ time”

 “Add some delays to the publication of papers” to “Delays the publication”

 “Reviewers’ databases”

 “to oversight” to “to oversee”

 “Augment the peer process” to “Augment the review process”

 “are not Involved” to “are not involved”

**Response:**

**Done! Thank you for being meticulous here.**

**Reviewer #2, continued:**

 Rephrase the subtitle, “Toward the promotion of Professional Reviewer as a career” to “Promoting professional reviewing as a career”. A review cannot be factually (logically) promoted as a career.

**Response:**

**Thank you for your suggestion. I have changed the subheading accordingly.**

**Reviewer #2, continued:**

 Please rephrase sentences that use dummy subjects, “it is,” “there is,” or “there are” as suggested by the principles of plain English and for the sake of brevity.

**Response:**

**Thank you. I have removed many instances of “it is” and “there is”.**

**Reviewer #2, continued:**

 Please avoid personifications, for example, “… this paper reckons …” while the credit should go to the author.

**Response:**

**This is now replaced by “I reckon …”. Thank you**

**Reviewer #2, continued:**

 Avoid using the passive voice and nominalizations (using verbs as nouns requiring helper verbs). For example, “Many calls have been made to reform …”

**Response:**

**I have now reduced the occurrence of passive forms (has/have been). Thank you.**

**Reviewer #2, continued:**

 Avoid contractions in academic writing, e.g., “I’m.”

**Response:**

**Done! Thank you.**

**Reviewer #2, continued:**

 Correct the minor spelling mistakes:

 ‘chose’ [choose in two places]

 ‘through evaluation’ [thorough evaluation]

 between journals [among journals]

 add a question mark after ‘Is the ultimate …’,

 ‘will soon or later’ [will sooner or later] …

**Response:**

**Done! Thank you.**

**Reviewer #2, continued:**

 The paper will benefit from proofreading, editing, and summarization.

 Thank you for the opportunity to review your work.

**Response:**

**I have edited the manuscript to correct any typing or grammatical errors. Thank you again for all these very useful and constructive comments and suggestions.**

# Reviewer report: Reviewer response for version 1

**DOI:** 10.5256/f1000research.163380.r277016

**Published:** 2024-05-31

**Authors:** Balazs Aczel

**Affiliations:** 1 Eotvos Lorand University, Budapest, Hungary

All in all, I found that the manuscript discusses an important and timely topic along a well structured argument. 

 My only major issue with the message of the paper is that it proposes paying referees as a solution to the peer review crisis although there are a number of listed and unresolved issues with the ideas. For example, the author shortly discusses that if paying reviewers becomes a norm then they might become uninterested in reviewing for free. This would be a very negative development for not-for-profit publishers, further strengthening the for-profit model that the author criticises. The author suggests that the budget for paying the reviewers could come from $200 submission fees which could be reduced from the article processing charges. Although it sounds like a fair solution, it would go against the publishers' financial interest since they would "lose" that income. From a profit-oriented point-of-view, it either makes the proposal sound unsavory unless the loss can be compensated by further increasing the APCs.

 Minor issues:

 - " exponential increase in the number of submissions", "desk rejection rates in reputable journals are reaching high rates" are factual statements without reference to evidence.

 - Among the potential solution could be mentioned PCI https://peercommunityin.org/ where the community organizes the reviews without any involvement of the journals. This model should reduce redundancies in the peer review system.

Is the topic of the opinion article discussed accurately in the context of the current literature?

Yes

Are arguments sufficiently supported by evidence from the published literature?

Yes

Are all factual statements correct and adequately supported by citations?

Yes

Are the conclusions drawn balanced and justified on the basis of the presented arguments?

Yes

Reviewer Expertise:

metascience

I confirm that I have read this submission and believe that I have an appropriate level of expertise to confirm that it is of an acceptable scientific standard.

# Response

**Published:** 2024-06-25

**Authors:** Mohamed Seghier

**Affiliations:** Khalifa University, United Arab Emirates

**Point-by-point responses:**

**I would like to thank both reviewers for their very helpful and constructive comments. All their concerns have been addressed in this resubmitted revision.**

**All changes are highlighted in the revised manuscript** **with track changes.**

**Below, is a detailed point-by-point rebuttal addressing each reviewer’s comment.**

**Reviewer #1:** Balazs Aczel, Eotvos Lorand University, Budapest, Hungary.

 All in all, I found that the manuscript discusses an important and timely topic along a well-structured argument.

**Thank you very much for your positivity. I’m happy to read that you did find the current version important and well-structured.**

**Reviewer #1, continued:**

 My only major issue with the message of the paper is that it proposes paying referees as a solution to the peer review crisis although there are a number of listed and unresolved issues with the ideas. For example, the author shortly discusses that if paying reviewers becomes a norm then they might become uninterested in reviewing for free. This would be a very negative development for not-for-profit publishers, further strengthening the for-profit model that the author criticises.

**Response:**

**Thank you for raising this important point. I have now added the following statement to clarify this issue on Page 10: “** ***There is also the risk that paying referees will soon become the norm, making researchers no longer interested in reviewing for free. It could also lead to a multi-tier peer review system depending on how much a journal chooses to pay its reviewers, hence engendering an unequal competition among journals for the service of reviewers. This could hurt not-for-profit publishers that cannot afford an expensive peer review process. In that context, not-for-profit publishers must (i) diversify incentives on top of paying reviewers, (ii) nurture the intrinsic motivation of their readers to participate in peer review and serve their scientific community*** ***[67]*** ***, (iii) seek alternative means to be subsidized*** ***[69]*** ***, and (iv) foster strategic partnerships with all stakeholders to make not-for-profit journals the preferred model for open-access publicly-funded research.*** ***Overall, although one*** ***cannot rule out the risk of corruption in peer review by money, I trust that academia has built-in mechanisms to handle financial transactions fairly and objectively, similar to cases when researchers are paid to evaluate grant proposals and academic theses*** ***[53]*** ***.*** **”**

**Reviewer #1, continued:**

 The author suggests that the budget for paying the reviewers could come from $200 submission fees which could be reduced from the article processing charges. Although it sounds like a fair solution, it would go against the publishers' financial interest since they would "lose" that income. From a profit-oriented point-of-view, it either makes the proposal sound unsavory unless the loss can be compensated by further increasing the APCs.

**Response:**

**I agree with the reviewer here. Accordingly, I have made the following changes:**

**To Page 8, I have added: “ *However, this might sound unsavory for for-profit publishers and hence the community needs to create alternative models that would make publishers active contributors to peer review, as discussed below.*”**

**See also the new edited text in the section “ *What about the publishers?*” (Page 9-10).**

**Reviewer #1, continued:**

 Minor issues:

 - " exponential increase in the number of submissions", "desk rejection rates in reputable journals are reaching high rates" are factual statements without reference to evidence.

**Response:**

**Thank you for spotting these missing citations. I have now added the following references:**

***Bornmann L, Haunschild R, Mutz R (2021) Growth rates of modern science: a latent piecewise growth curve approach to model publication numbers from established and new literature databases. Humanities and Social Sciences Communications, 8:224.***

***Larsen PO, von Ins M (2010) The rate of growth in scientific publication and the decline in coverage provided by Science Citation Index. Scientometrics, 84:575-603. 10.1007/s11192-010-0202-z.***

***Meyer HS, Durning SJ, Sklar DP, Maggio LA (2018) Making the First Cut: An Analysis of Academic Medicine Editors' Reasons for Not Sending Manuscripts Out for External Peer Review. Acad Med, 93:464-470. 10.1097/ACM.0000000000001860.***

**Reviewer #1, continued:**

 - Among the potential solution could be mentioned PCI https://peercommunityin.org/ where the community organizes the reviews without any involvement of the journals. This model should reduce redundancies in the peer review system.

**Response:**

**Thank you for this very important suggestion. I have now added PCI to Table 1. Please see the new revised version of the resubmitted manuscript.**
